# Comparison of metagenomic samples using sequence signatures

**DOI:** 10.1186/1471-2164-13-730

**Published:** 2012-12-27

**Authors:** Bai Jiang, Kai Song, Jie Ren, Minghua Deng, Fengzhu Sun, Xuegong Zhang

**Affiliations:** 1 MOE Key Laboratory of Bioinformatics, Bioinformatics Division and Center for Synthetic and Systems Biology, TNLIST / Department of Automation, Tsinghua University, Beijing 100084, China; 2 School of Mathematical Sciences, Peking University, Beijing 100871, China; 3 Molecular and Computational Biology, University of Southern California, Los Angeles, CA 90089, USA; 4 School of Life Sciences, Tsinghua University, Beijing 100084, China

## Abstract

**Background:**

Sequence signatures, as defined by the frequencies of *k*-tuples (or *k*-mers, *k*-grams), have been used extensively to compare genomic sequences of individual organisms, to identify *cis*-regulatory modules, and to study the evolution of regulatory sequences. Recently many next-generation sequencing (NGS) read data sets of metagenomic samples from a variety of different environments have been generated. The assembly of these reads can be difficult and analysis methods based on mapping reads to genes or pathways are also restricted by the availability and completeness of existing databases. Sequence-signature-based methods, however, do not need the complete genomes or existing databases and thus, can potentially be very useful for the comparison of metagenomic samples using NGS read data. Still, the applications of sequence signature methods for the comparison of metagenomic samples have not been well studied.

**Results:**

We studied several dissimilarity measures, including *d*_*2*_, *d*_2_^*^ and *d*_2_^*S*^ recently developed from our group, a measure (hereinafter noted as *Hao*) used in CVTree developed from Hao’s group (Qi *et al*., 2004), measures based on relative di-, tri-, and tetra-nucleotide frequencies as in Willner *et al*. (2009), as well as standard *l*_*p*_ measures between the frequency vectors, for the comparison of metagenomic samples using sequence signatures. We compared their performance using a series of extensive simulations and three real next-generation sequencing (NGS) metagenomic datasets: 39 fecal samples from 33 mammalian host species, 56 marine samples across the world, and 13 fecal samples from human individuals. Results showed that the dissimilarity measure *d*_2_^*S*^ can achieve superior performance when comparing metagenomic samples by clustering them into different groups as well as recovering environmental gradients affecting microbial samples. New insights into the environmental factors affecting microbial compositions in metagenomic samples are obtained through the analyses. Our results show that sequence signatures of the mammalian gut are closely associated with diet and gut physiology of the mammals, and that sequence signatures of marine communities are closely related to location and temperature.

**Conclusions:**

Sequence signatures can successfully reveal major group and gradient relationships among metagenomic samples from NGS reads without alignment to reference databases. The *d*_2_^*S*^ dissimilarity measure is a good choice in all application scenarios. The optimal choice of tuple size depends on sequencing depth, but it is quite robust within a range of choices for moderate sequencing depths.

## Background

The study of dissimilarity between samples, known as beta-diversity (***β***-diversity), is crucial for studying microbial communities from environmental or human niches [1]. Beta-diversity gives a quantitative measure of differences between two microbial samples, forming the basis for a quantitative comparison of multiple samples. The dissimilarity matrix defined by beta-diversity measures between all pairs of samples in a set of multiple samples can be utilized to study group relationships of the samples [2] and to understand environmental gradients affecting microbial samples [3].

Valuable insights into the diversity of hundreds of uncultured microbial samples from various environments or niches have been provided by sequencing the small subunits of ribosomal RNA, specifically the 16S rRNA, with the conventional Sanger sequencing or the next-generation sequencing (NGS) technologies from a variety of environments, including soil [4,5], ocean [6-8], mammalian gut [9], human skin [10-12], human gut [13-15], and human oral cavity [16-18], among many others. In 16S rRNA-based surveys, several analytical procedures have been carried out to compare multiple microbial samples using different beta-diversity measures [19]. The two general categories of beta-diversity measures include phylogenetic- and taxon-based methods. Using UniFrac [20-23] as an example, phylogenetic-based methods first generate a phylogenetic tree of sequences in each sample and then compare samples by counting overlaps of branches of their corresponding trees. Taxon-based methods, on the other hand, calculate beta-diversity through binning sequences to Operational Taxonomic Units (OTUs), or assigning sequences to, for example, species or genera, and then comparing samples by counting overlaps in the taxa [24-26].

With the rapid development of NGS technology, whole metagenome shotgun sequencing (WMGS) is becoming a new powerful approach to investigate complex microbial samples [27-31]. Metagenomics data provide more complete information on the microbial community, but beta-diversity measures for metagenomic sequencing reads from different microbial communities are significantly under-studied. A common practice of analyzing metagenomics data is to first map the short reads to known genes or pathways in existing databases, such as NR, KEGG or IMG and then compare their abundances between samples based on the mapped functional categories (e.g., [31]). However, as a result of the incompleteness of microbial genomic annotation and function databases, only a small fraction of reads can be mapped to known genes and pathways, resulting in significant loss of information in the comparison of metagenomes.

Genome sequence signatures refer to the frequencies of *k*-tuples (*k*-mers, *k*-grams) in a genome. Previous studies have shown that *k*-tuple frequencies are similar across different regions of the same genome, but differ between genomes [32]. Sequence signatures have been widely used to study the evolutionary relationships among genomic sequences [33,34], to study horizontal gene transfer among different genomes [35] and to bin genome fragments from metagenomic samples [36,37]. In metagenomic studies, the number of organisms in the communities, the complete genome sequences of the organisms, and their abundance levels are usually not known. Thus, samples cannot be directly compared on the basis of abundance levels of organisms within communities. Methods based on mapping reads to known genes or functional categories are also restricted by the availability and completeness of existing databases. However, since genomes have their sequence signatures, differences in microbial compositions of samples will result in differences in sequence signatures of the metagenomes.

Thus far, sequence signatures have not been widely applied in the quantitative comparison of metagenomic samples, except a few studies that used di-, tri-, and tetra-nucleotide signatures (e.g., [38,39]). On the other hand, with the availability of NGS data, sequence-signature-based methods have high potential for the comparison of metagenomes. NGS technologies have provided efficient approaches to sample short reads of DNA sequences from the metagenomic communities. Maillet et al. [40] recently presented an algorithm to efficiently find similar reads between two metagenomic datasets based on *k-*tuple signatures. Without using complete genomes or information of any known genes, sequence signatures of the metagenomes can be reflected from the NGS short reads and can be easily calculated. Thus, we can use sequence signatures to compare metagenomic samples from NGS short reads without doing sequence assembly or alignment.

Key steps in sequence signature methods include the counting of frequencies of all *k*-tuples to compose the sequence signature vector for each sample, calculating the dissimilarity measure between samples based on their sequence signature vectors, and analyzing relationships between multiple samples based on dissimilarities of all sample pairs. In this study, we systematically investigated these key steps using a series of simulated and real metagenomic datasets, giving special attention to the choice of dissimilarity measures, the length of the sequence signatures, and the model for the background genome sequences for the comparison of multiple metagenomic samples.

## Results

We conducted a series of computational experiments by both extensive simulations and real data analyses to study the effectiveness of the sequence signature methods in identifying group and gradient relationships of microbial community samples. We first simulated four types of datasets to investigate factors that may affect the performance of the sequence signature methods. The simulated datasets were generated by sampling from mixtures of multiple true genomes with different abundance levels. We studied three *d*^*2*^*−*type dissimilarity measures, as defined in [34], based on *k*-tuple count vectors and three dissimilarity measures defined on the basis of comparing the actual *k*-tuple frequency vectors to evaluate the beta-diversity between different samples. We also studied the performance of a dissimilarity measure used in CVTree (*Hao*) [41] and measures based on di-, tri-, and tetra-nucleotide signatures (*Willner*) [38]. To calculate *d*_2_^*S*^ and *d*_2_^*^, the expected count of the *k*-tuples under a suitable probability model for the background sequences is needed. Here Markov models of different orders for the underlying background genomes were used to calculate the expected counts of *k*-tuples. We studied a total of 14 dissimilarity measures (see Materials and Methods for details). Using the dissimilarity matrix obtained from any one of the measures, we studied the relationships of the samples.

### Simulation 1: detecting group relationships among metagenomic samples of relatively low complexity

In some situations, metagenomic samples may form different groups. For example, gut samples may group based on diet, and marine samples may group based on locations. In order to evaluate the ability of the dissimilarity measures to detect such group relationships, we simulated datasets of 90 metagenomic samples belonging to 3 different groups (30 samples in each group) similar to the simulation method of Kuczynski *et al*. [19]. Each sample was generated by simulating NGS short reads from a mixture of the genomes of 5 microbial species detected in soil [42] with different abundance levels (see Materials and Methods for details).

We applied the 14 dissimilarity measures based on sequence signatures (defined in Materials and Methods) with different *k*-tuple sizes *k* = 2--10 in this dataset to detect the group relationships of the 90 samples by clustering analysis. For a specific tuple size *k*, *k*-tuple count/frequency vector of each sample was first calculated, and samples were compared with each other using each dissimilarity measure. We then used the UPGMA method [43] to cluster the samples based on the dissimilarity matrix. The parsimony test [25] was finally used to compare the derived clusters with the actual groups given in the simulation model.

**Figure 1:**
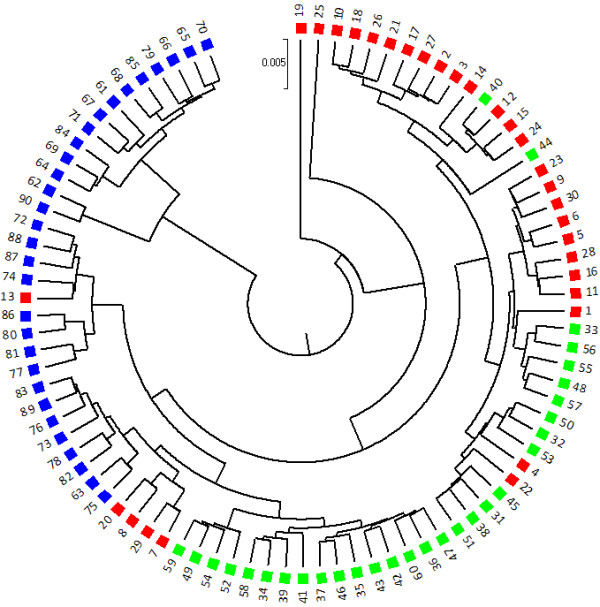
**Metagenomic samples can be clustered well using dissimilarity measure *d***_**2**_^***S***^|***M***_**0. **_Example of a clustering of 90 samples in Simulation 1 obtained at sequencing depth = 10,000 reads per sample, with *k *= 6 and dissimilarity measure *d*_2_^*S*^|*M*_0_. The true clusters are labeled by three colors and symbols (red triangles, green squares and blue discs). The parsimony test score is 10 in this example with *p-*value less than 0.001.

The number of sequences in each sample, termed sequencing depth, may affect the accuracy of the sequence signature methods. We simulated three different sequencing depths: 1,000, 10,000, and 100,000 reads per sample. To study the stochastic variation of the results, the simulation was repeated 100 times at each sequencing depth. As an example, Figure 1 shows one clustering result of the 90 samples at sequencing depth of 10,000 obtained with *k* = 5 and the dissimilarity measure *d*_2_^*S*^|*M*_0_, which is the *d*_2_^*S*^ dissimilarity measure (Eq.2) under the Markov model of order 0 *(M*_*0*_*)*, the independent identically distributed (i.i.d.) model. It can be seen that most of the samples in the original 3 groups can be clustered together. Compared with random clustering results, the parsimony test score is highly statistically significant with Monte Carlo *p*-value <<0.001.

**Figure 2:**
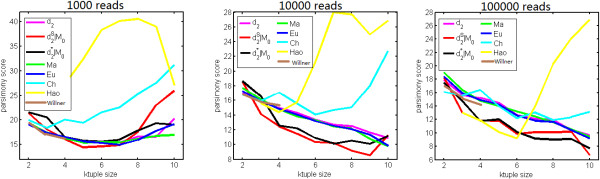
**The relative performance of various dissimilarity measures at difference sequencing depths in recovering group relationships of the metagenomic samples.** The dissimilarity measures *d*_2_^*^ and *d*_2_^*S *^outperform others in most situations.

We studied various factors, including the order of the Markov model for the background sequence, the tuple size *k*, and sequencing depth, affecting the performance of *d*_2_^*S*^ and *d*_2_^*^ in recovering the group relationships of the samples. Figure 2 shows the relative performance of *d*_2_^*S*^ and *d*_2_^*^ coupled with the i.i.d. model for the background sequence with other dissimilarity measures, including *d*_*2*_, *Ma*, *Eu*, *Ch*, *Hao*, and *Willner*. Overall, the dissimilarity measures *d*_*2*_, *Ma*, *Eu*, *Ch*, *Hao* and *Willner* do not perform as well as *d*_2_^*S*^ and *d*_2_^*^. The poor performance of *d*_*2*_ can be explained by the fact that it is dominated by the variation of the tuple occurrences within one sample, and is less affected by the relationship between the sequences in both samples. Meanwhile, the poor performance of *Hao* could be attributed to the high number of parameters that need to be estimated to fit a Markov model of order *k*-2. *Ch* considers the maximum difference between the tuple frequencies for the samples only and does not make full use of the information from all the tuples. Most importantly, the normalization of the tuple counts plays an important role in the superior performance of *d*_2_^*S*^ and *d*_2_^*^. For each dissimilarity measure, the optimal tuple size giving the lowest parsimony score depends on the sequencing depth. For example, when the sequencing depth is 1,000, the optimal tuple size for *d*_2_^*S*^, *d*_2_^*^, *Eu*, and *Ma* is around 5 to 7. When sequencing depth is high (100,000 reads or higher), the parsimony score for *d*_2_^*S*^, *d*_2_^*^, *Ma*, and *Eu* decreases as the tuple size increases from 2 to 10.

Next we studied the effect of the order of the Markov model for the background sequences on the parsimony score of *d*_2_^*S*^ and *d*_2_^*^, and the results are given in Additional file 1: Figure S1. When the sequencing depth is low (< 10,000 reads), the i.i.d. model gives the lowest parsimony score in general. When the sequencing depth is high (>100,000 reads), the effect of the order of the Markov model is negligible and the results are similar across the different orders (0, 1, 2, and 3), especially when the tuple size is relatively large (*k* > 6). One potential explanation for the above observations is as follows. When the sequencing depth is low, the absence of sufficient data to accurately estimate the many parameters in high-order Markov models results in the low accuracy to cluster the metagenomic samples. However, when the sequencing depth is high, this is not a problem and Markov models of different orders give similar results.

Finally, we studied the standard deviations of the parsimony score of *d*_2_^*S*^ and the results are given in Additional file 1: Figure S2. From the figure we can see that at lower sequencing depth (1,000 sequences), the best performance is obtained at *k* = 5. When the sequencing is 10 times deeper, the best *k* becomes 9, and it further increases when the sequencing depth further increases. We can also see that the standard deviation of the parsimony score among the 100 repeated experiments at each sequencing depth is quite moderate and stable with different choices of *k*, and deeper sequencing will decrease the standard deviation of the parsimony scores, as expected.

### Simulation 2: revealing environmental gradients from metagenomic samples of relatively low complexity

The second simulation experiment was designed to evaluate the effectiveness of sequence signature methods for analyzing gradient variation of microbial communities. A set of 20 metagenomic samples was generated by simulating NGS reads from 5 soil bacterial species as in the above simulations [42] with varying abundance levels. We designed the proportion of the 5 genomes to vary from sample 1 to sample 20 in a way that mimics the situation of gradient variation across the samples, and we then studied how well the sequence signature methods reveal such gradient variations from the metagenomic data. Dissimilarity matrices were calculated using different dissimilarity measures and different *k*-tuple sizes as above. PCoA [44], an effective approach to display beta-diversity among multiple samples, mapped the 20 samples to a two-dimensional space. Then the Pearson Correlation Coefficient (PCC) was calculated between the first principal coordinate (PC1) given by PCoA and the predetermined gradient axis built in the simulation model. The PCC can be taken as an index of how well the sequence signature method reveals the gradient variation in samples (see Materials and Methods for details). A higher PCC indicates better performance of the dissimilarity measure in recovering the gradient among the microbial samples.

**Figure 3:**
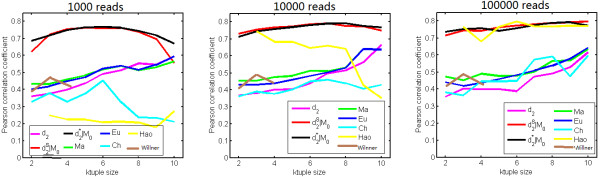
**The relative performance of various dissimilarity measures at different sequencing depths in recovering gradient relationships of metagenomic samples.** The dissimilarity measures *d*_2_^*^ and *d*_2_^*S *^outperform others in most situations.

Similar to Simulation 1, we repeated the simulation experiments 100 times at each of the three sequencing depths of 1,000, 10,000 and 100,000 reads per sample. Figure 3 shows the average PCC of the different dissimilarity measures at different sequencing depths and tuple sizes. We can see that the dissimilarity measures perform with greater differences than observed in the case of group relationships in Simulation 1. Specifically, the performance of *d*_2_^*S*^ and *d*_2_^*^ is quite robust with respect to the tuple size and the sequencing depth. In most situations, *d*_2_^*S*^ and *d*_2_^*^ perform similarly manner, and outperform other dissimilarity measures. The average PCCs for these two measures are larger than 0.75 when tuple size *k* is between 4 and 8, and the sequencing depth is 10,000. Deeper sequencing can slightly increase the PCC but the improvement is not as significant as that observed in Simulation 1. Additional file 1: Figure S3 shows that the performance of *d*_2_^*S*^ and *d*_2_^*^ is highly robust with respect to the order of Markov model used to calculate the expected occurrences of *k*-tuples. Additional file 1: Figure S4 shows the means and standard deviations of PCC obtained using the dissimilarity measure *d*_2_^*S*^ with tuple sizes k = 2--10 at different sequencing depths. We can see that the optimal tuple sizes, at lower or moderate sequencing depths, are between 4 and 7, and this range also gives the smallest standard deviation although the standard deviation is, in general, very small.

### Simulations 3 and 4: revealing group relationship and environmental gradients from metagenomic samples of relatively high complexity

The above simulation experiments illustrated the performance of the various dissimilarity measures for microbial communities of relatively low complexity. Most microbial communities are much more complex with hundreds to thousands of genomes. In order to see the performance of the dissimilarity measures for discovering group or gradient relationships among metagenomic samples of high complexity, we simulated two NGS metagenomic datasets with more complex microbial compositions. The simulated communities consist of 113 microbial genomes from a collection of genomes given by the FAMeS dataset [45,46]. We simulated short reads with both Roche/454 and Illumina/Solexa platforms.

In Simulation 3, we generated 60 samples belonging to 3 groups, as defined by different compositions of the microbial genomes (see Materials and Methods for details). In Simulation 4, we simulated 20 samples related through a gradient (see Materials and Methods for details). As in Simulations 1 and 2, we generated datasets at three sequencing depths: 1,000, 10,000 and 100,000 reads per sample. For each setting, we generated 100 duplicated datasets to simulate possible stochastic effects in real NGS data.

The complete simulation results are given in Additional file 1: Figures S5-S7. The qualitative results for the relative performance of the different dissimilarity measures are similar to that from the first two simulations for metagenomic samples of relatively low complexity. We also changed the read length for Roche/454 and Illumina/Solexa in our simulations and the results reported above continue to hold.

### Detecting group relationships among mammalian gut samples

We applied the sequence signature methods to analyze a real mammalian gut metagenomic dataset by Muegge *et al*. [31]. It includes the NGS short reads of 39 metagenomic samples from 33 mammalian species of herbivores, carnivores and omnivores (Additional file 1: Table S1). Previous studies showed that the microbial compositions of omnivores are very diverse and cannot be distinguished from other samples [31]. Consequently, we first excluded the 11 omnivore samples and focused on the remaining 28 herbivore and carnivore samples. These samples include 3 groups: hindgut-fermenting herbivores (*n* = 8), foregut-fermenting herbivores (*n* = 13), and simple-gut carnivores (*n* = 7). We investigated how well the sequence signature methods identified these 3 groups from the NGS metagenomic data. Similar to the simulation studies, we used UPGMA to cluster the samples based on the dissimilarity matrix, as defined by different dissimilarity measures based on sequence signatures, and we assessed how well the resulting cluster tree revealed the underlying 3 groups by the parsimony test.

The resulting parsimony scores for the different dissimilarity measures and tuple size are summarized in Table 1. For most choices of the tuple size *k* and dissimilarity measures, the sequence signature methods could group the samples well. This indicates significant group differences in sequence signatures of the metagenomes among hindgut-fermenting herbivores, foregut-fermenting herbivores and simple-gut carnivores. The smallest parsimony score (= 2, indicating best grouping of the samples) was achieved with *d*_2_^*S*^ coupled with the i.i.d. model for the background sequences (M_0_)and *k* = 5. When *k* varies between 3 and 9, the parsimony scores obtained with *d*_2_^*S*^|*M*_0_ are never greater than 4 and are always the smallest among those with other dissimilarity measures. This shows that *d*_2_^*S*^|*M*_0_ has the best performance among all the dissimilarity measures we considered and that the optimal performance is not very sensitive to the choice of tuple size *k* as long as it is within a reasonable range. When the tuple size *k* was increased to 10 or above, we also observed that the performances of all dissimilarity measures became worse. To explain, with a given data size and large *k*, the number of occurrences of each *k*-tuple will be too small, causing the result to be unstable. We also experimented with *k* = 2, which also resulted in poor performances. However, when *k* = 2, the sequence signature vector only has 16 dimensions and therefore cannot adequately reflect the diversity of the samples.

**Table 1:** Parsimony scores on the clustering of the three mammalian groups (hindgut-fermenting herbivores, foregut-fermenting herbivores and simple-gut) obtained by sequence signature methods using different dissimilarity measures based on the metagenomic data

*k*	2	3	4	5	6	7	8	9	10
*d*_*2*_	7	5	5	5	5	5	6	6	5
*d*_2_^*S*^|*M*_0_	5	**3**	**3**	**2**	4	4	4	4	6
*d*_2_^*S*^|*M*_1_	9	4	**3**	4	4	4	4	5	7
*d*_2_^*S*^|*M*_2_	NA	4	**3**	5	4	4	4	4	6
*d*_2_^*S*^|*M*_3_	NA	NA	**3**	4	4	5	4	4	7
*d*_2_^*^|*M*_0_	7	7	6	4	5	4	4	6	7
*d*_2_^*^|*M*_1_	8	5	4	4	5	5	5	6	7
*d*_2_^*^|*M*_2_	NA	4	5	5	5	5	6	6	7
*d*_2_^*^|*M*_3_	NA	NA	4	4	5	5	5	5	6
*Ma*	7	5	5	5	5	5	5	4	6
Eu	7	5	5	5	5	5	5	7	5
Ch	7	6	5	5	5	4	5	6	7
Hao	NA	5	4	4	6	6	8	9	11
Willner	11	10	8						

The “M_i_” indicates that the expected counts are calculated based on *i*-th order Markov model for the background sequences. Monte Carlo *p*-values were estimated by comparing observed parsimony score to the scores in 1000 randomly-joined trees: parsimony score = 2--7, p < 0.001; score = 8, p = 0.002. Boldfaced are the two lowest parsimony scores.

**Figure 4:**
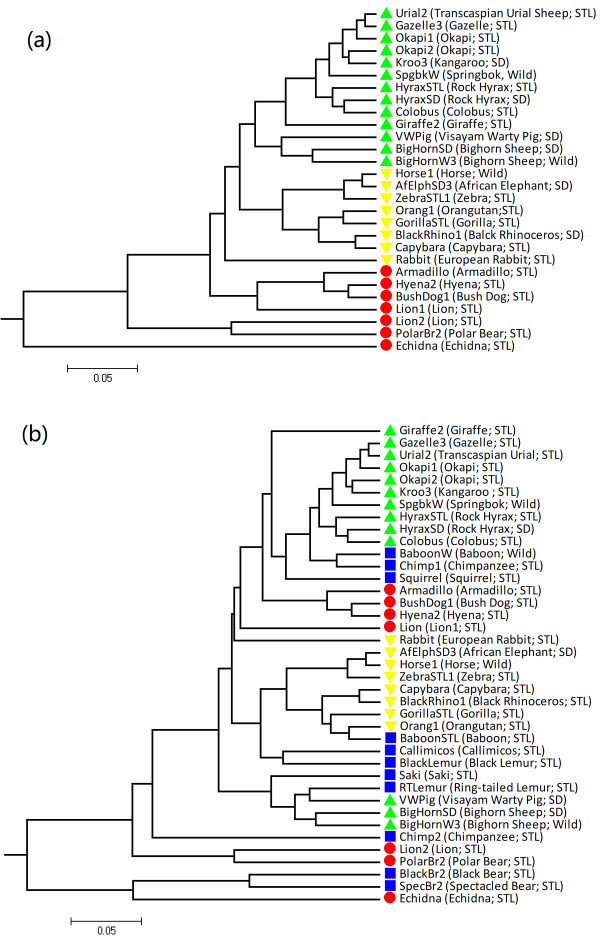
**Clustering results of the mammalian gut samples based on 5**-**tuples and dissimilarity measure *d***_**2**_^***S***^|***M***_**0**_**: (a) without the omnivore samples; (b) with the omnivore samples.** Green upward triangles: foregut-fermenting herbivore; yellow downward triangles: hindgut-fermenting herbivore; red discs: simple-gut carnivore; blue squares: simple-gut omnivore.

Figure 4a shows the clustering tree obtained with tuple size *k* = 5 and *d*_2_^*S*^|*M*_0_. Clear separations among the three groups of samples can be observed. The relationships of the samples can also be visualized in 2-dimension by the PCoA plot in Additional file 1: Figure S8(a). From the PCoA plot, we can see that 3 of the 4 pairs of samples with the same host species are also clustered together: 2 Okapis (Okapis1 and Okapis2), 2 Bighorn Sheeps (BigHorn1 and BigHorn2) and 2 Rock Hyraxes (HyraxSD and HyraxSTL), while the 2 Lions (Lion1 and Lion2) are separated. Moreover, the digestive physiology of the host samples (foregut-fermenting vs. hindgut-fermenting) is the most distinguishing information extracted by the sequence signature method. This observation was not reported from this dataset in the original study [31].

Then we added back the 11 omnivorous samples to the dataset and reanalyzed the data, and the result is shown in Figure 4b. We can see that the omnivore samples are not grouped as a single cluster, but are scattered throughout the clustering tree. This suggests more diversity in the fecal microbiome of omnivores than that of herbivores or carnivores, consistent with previous observations based on 16S rRNA data [31]. Some omnivore samples are clustered together with samples of other diets. For example, samples from omnivorous bears (Spectacled Bear, SpecBr2 and Black Bear, BlackBr2) share considerable similarity with those from carnivores, as previouslyreported in [9]. The PCoA plot including the omnivore samples is given in Additional file 1: Figure S8(b).

### Detecting group and gradient variations in global ocean metagenomics data

We applied the sequence signature methods to analyze the metagenomics data of global ocean samples collected from different geographic locations and conditions by Rusch *et al*. [29]. The geographic locations of the samples include the Sargasso Sea, Caribbean Sea, Eastern Tropical Pacific and Tropical South Pacific, and the samples also contain variation in microbial habitat types (e.g., open and coastal) and environmental temperatures (north temperate, south tropical, and north tropical) (see Additional file 1: Table S2). All these factors may influence the composition of seawater microbiomes. To avoid the interacting effect of locations and habitat types, we applied the sequence signature methods on the 23 open ocean samples and the 19 coastal water samples separately.

**Figure 5:**
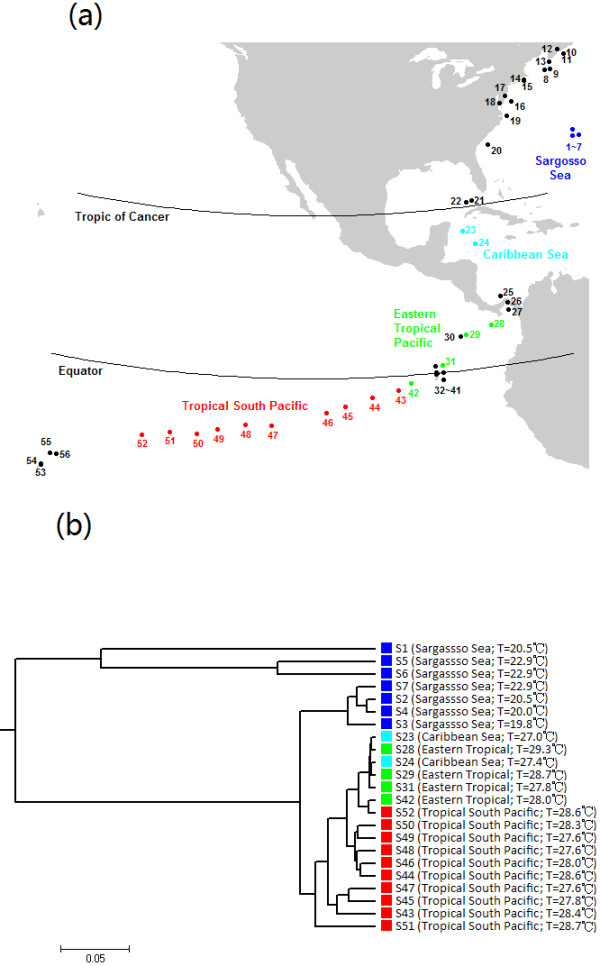
**Cluster analysis of open ocean samples from different geographical locations.(a) **Geographical locations of the 56 global ocean samples. The 23 open ocean samples are indicated as follows: Sargasso Sea (n=7, blue), Caribbean Sea (n=2, cyan), Eastern Tropical Pacific (n=4, green), and Tropical South Pacific (n=10, red). **(b) **Clustering results of open ocean samples based on 5-tuples and dissimilarity measure *d*_2_^*S*^|*M*_0_.

The 23 open ocean samples form four geographic groups: Sargasso Sea (*n* = 7), Caribbean Sea (*n* = 2), Eastern Tropical Pacific (*n* = 4), and Tropical South Pacific (*n* = 10) (Figure 5a). We conducted clustering analysis with sequence signatures on these samples and used the parsimony test to study how well the grouping information was revealed (Table 2). Again, for most tuple size values, it can be seen that *d*_2_^*S*^|*M*_0_ achieves the lowest parsimony score among all the dissimilarity measures we studied, and also the clustering results are quite stable when the tuple size *k* is between 5 and 9. Figure 5b shows the clustering tree of the open ocean samples with *k* = 5 and *d*_2_^*S*^|*M*_0_. We observed that the three major groups identified by the sequence signature method reflect three major environmental temperature conditions: north temperate, north tropical, and south tropical. The samples from the Caribbean Sea were clustered together with those from the Eastern Tropical Pacific Ocean. They both lie between the Tropic of Cancer and the Equator.

**Table 2:** Parsimony scores on the clustering tree of 23 open ocean samples belonging to 4 groups (Sargasso Sea, Caribbean Sea, Eastern Tropical Pacific and Tropical South Pacific) obtained by sequence signature methods using different dissimilarity measures based on the open ocean metagenomics data

*k*	2	3	4	5	6	7	8	9	10
*d*_*2*_	6	6	6	7	7	7	7	7	6
*d*_2_^*S*^|*M*_0_	5	6	5	**4**	**4**	**4**	**4**	**4**	5
*d*_2_^*S*^|*M*_1_	11	6	5	6	5	5	6	5	**4**
*d*_2_^*S*^|*M*_2_	NA	8	**4**	6	5	5	5	5	5
*d*_2_^*S*^|*M*_3_	NA	NA	5	6	6	5	6	5	5
*d*_2_^*^|*M*_0_	5	5	**4**	**4**	6	6	6	7	7
*d*_2_^*^|*M*_1_	9	7	7	5	6	6	6	7	7
*d*_2_^*^|*M*_2_	NA	8	7	5	5	5	7	6	7
*d*_2_^*^|*M*_3_	NA	NA	6	5	5	5	7	7	6
*Ma*	6	5	5	5	5	**4**	**4**	5	**4**
Eu	6	6	5	6	8	7	7	7	7
Ch	7	7	8	9	9	7	7	6	6
Hao	NA	5	6	6	6	6	**4**	5	6
*Willner*	7	6	5						

The “M_i_” indicates that the expected counts are calculated based on *i*-th order Markov model for the background sequences. Monte Carlo *p-*values were estimated by comparing observed parsimony score to the scores in 10000 random joined trees: parsimony score = 4--6, *p* < 0.001; score = 7, *p *= 0.004; score = 8; *p *= 0.003; score = 9; *p* = 0.12. Boldfaced are the two lowest parsimony scores.

We also found significant separation among 19 coastal water samples (see Additional file 1: Table S3), mainly consisting of 9 samples from the North American East Coast and 6 samples from the Galapagos Islands in the Eastern Tropical Pacific (Additional file 1: Figure S9(a)). In the clustering tree obtained with 5-tuples and *d*_2_^*S*^|*M*_0_, coastal water samples from the North American East Coast are separated from those from the Galapagos Islands (Additional file 1: Figure S9(b)). However, we also see some possible errors in the clustering. For example, sample 20 from the south of Charleston, SC, was clustered with samples from the Galapagos Islands, and sample 39 from the Galapagos Islands was clustered together with samples from the North American East Coast. These outliers deserve further investigation.

**Figure 6:**
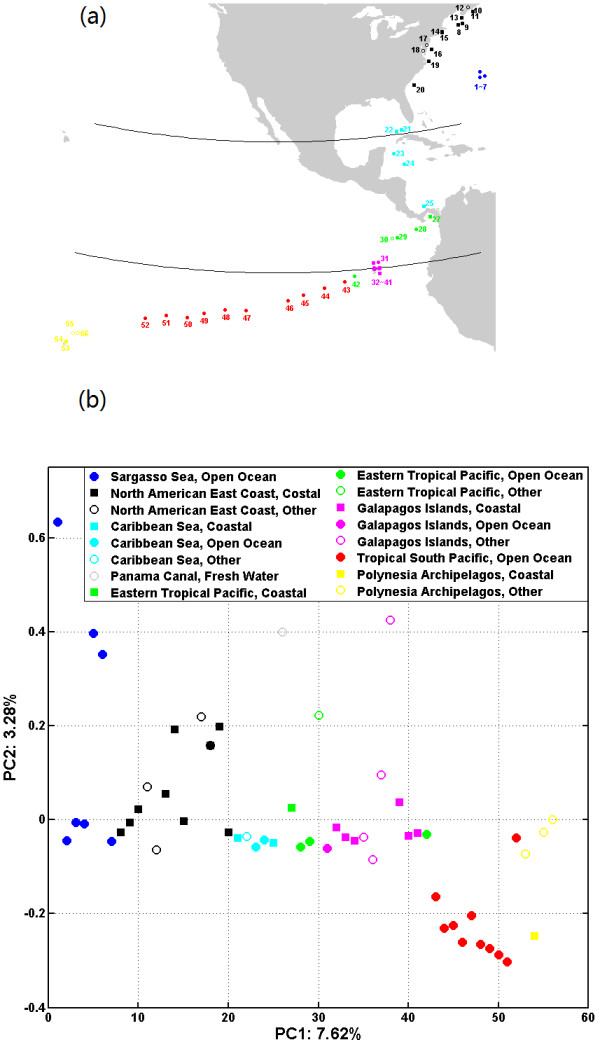
**PCoA ordinates of all 56 global ocean samples are primarily driven by geographical location. (a) **Geographic locations of all samples. **(b)** PCoA plot with *k *= 5 and dissimilarity measure *d*_2_^*S*^|*M*_0_. For the location, Sargasso Sea samples are colored blue, North America East Costal samples are colored black, Caribbean Sea samples are colored cyan, Eastern Tropical Pacific samples are colored green, Galapagos Islands samples are colored pink, Tropical South Pacific samples are colored red and Polynesian Archipelagos samples are colored yellow. For the habitat type, open ocean samples are marked as discs, coastal samples are marked as squares, and other habitat samples are marked with open circles.

We then pooled all 56 global ocean samples and carried out PCoA on all samples. Figure 6b gives the PCoA plot with *k* = 5 and *d*_2_^*S*^|*M*_0_. It can be clearly seen that geographic location as the gradient primarily drives the samples in the PCoA plot. Samples along the back-diagonal of the PCoA plot are geographically located along the S-shaped line sequentially running through the Sargasso Sea (blue), North American East Costal (black), Caribbean Sea (cyan), Eastern Tropical Pacific (green), Galapagos Islands (pink), Tropical South Pacific (red) and Polynesian Archipelagos (yellow). It is worth mentioning that sample 42 is both located and PCoA plotted between the Galapagos Islands (pink) and the Tropical South Pacific (red), although it belongs to the Eastern Tropical Pacific group (green). However, the habitats (open ocean: filled circle; coastal: filled square; other: open circle) do not significantly affect the clustering of the microbial communities.

### Experiments on the human gut metagenome data

We applied cluster analysis based on sequence signatures of the 13 human gut metagenome samples published by Kurokawa *et al*. [28]. One set of samples consists of4 unweaned infants, and the other consists of 7 adults and 2 weaned children [28]. The sample information is provided in Additional file 1: Table S4. Although the sample size is very small in this study, we asked whether sequence signature methods could still reflect meaningful relationships. Additional file 1: Table S5 gives the parsimony scores on the separation of these two groups by the clustering based on sequence signatures. Again, we see that *d*_2_^*S*^|*M*_0_ has the best performance for almost all values of tuple size *k*. The clustering tree and the ordination plots for the samples are shown in Additional file 1: Figure S10. It can be seen that the samples from the unweaned infants tend to cluster together but show more diversity than the adults and weaned children.

## Discussion

In this study, we examined the application of sequence signature methods for the comparison of microbial metagenomic samples with NGS read data. We studied both traditional and recently developed dissimilarity measures of sequence signature vectors, and we used Markov models of different orders to estimate the background when a measure requires a background model. The dissimilarity measures, with a wide range of choices of tuple size *k*, were compared on the basis of four sets of simulated metagenomics data and three sets of real metagenomics data, including metagenomes of gut samples of multiple mammalian species, water samples of global ocean, and human gut samples. The data represent a wide spectrum of topics that can be studied with metagenomic approaches.

In all the data we studied, sequence signature methods were able to reveal the major group and gradient variation among the samples from the short reads of metagenomic data. The performance of different dissimilarity measures varies with different choices of tuple size. The recently proposed *d*_2_^*S*^ measure performs the best in all scenarios and the optimal tuple size for it is between 5 and 9, for NGS data with moderate sequencing depth of about 10,000 sequences of 200nt. Its performance is not highly sensitive to the tuple size, making it easier to apply the method on real data. The experiments also show that new biological insights on factors affecting the *k*-tuple compositions of the metagenomes can be obtained. In the mammalian gut data experiment, the first factor is the diet, followed by the digestive physiology. For the global ocean samples, the most important factor is location, followed by habitat. For the human gut samples, weaning is a key factor that distinguishes microbiomes of adults and children from those of infants.

In summary, this work shows that the recently developed dissimilarity measure *d*_2_^*S*^ provides a powerful approach for metagenomic sample comparison based on NGS shotgun reads. It is simple and computationally efficient, and it can reveal underlying relationships between microbial community samples. The rapid development of NGS technology has provided great opportunities for metagenome sequencing. Because availability of known microbial genome references are limited and *de novo* assembly of metagenome sequences is challenging, the alignment-free sequence signature method is a promising approach for analyzing multiple metagenomic samples.

In this study, we concentrated on the comparison of metagenomic samples using alignment-free methods with sequence signatures. However, with respect to optimizing strategies for the comparison of metagenomic samples, several questions remain. First, we did not study how sequence signature methods compare with other methods using tag sequences such as 16S rRNA or its variable regions for microbiome comparison. Since the 16S gene is only a very small fraction of the metagenome, our prior experiences showed that extracting 16S sequences and classifying the samples using only the 16S gene sequences do not work well. Only using the 16S sequences of the metagenomic samples loses information, resulting in inaccurate clustering of the samples. Second, we do not know whether metagenome shotgun reads or assembled contig sequences should be used for metagenomic sample comparison. We did not conduct experiments to specifically address this question, but based on our experiments on the shotgun reads, sequence signatures can be extracted reasonably well with NGS short reads without any assembly. Third, for most metagenomics studies, investigators are interested in what genes or pathways are active. To achieve this objective, reads are usually mapped to the known genes or pathways, and then the samples are compared using the active genes or pathway. Such approaches can give important insights about functions of genes and pathways, which sequence signature-based approaches cannot. On the other hand, not all genes/pathways are known, and typically only a relatively small fraction of the reads can be mapped to known genes/pathways. Therefore, such gene/pathway-based approach cannot make full use of the data for metagenome comparison. Sequence signature-based methods use all reads and can potentially reveal more accurate relationships of samples. Finally, all our experiments were based on real datasets with all the potential complexities, such as sequence errors, heterogeneous sampling of reads from different organisms or different genomic regions, as well as biases arising from different techniques for sample preparation and sequencing. Despite all these potential complexities, it is surprising that the clustering of samples using sequence signatures with the *d*_2_^*S*^ measure still performs very well. Theoretical and further simulation studies to understand the effects of such complexities on the performance of different dissimilarity measures will be a topic for future research. The code for calculating the three d_2_-type measures is provided at http://www-rcf.usc.edu/fsun/Programs/d2Meta/d2Metamain.html.

## Conclusions

With the rapid development of NGS technologies and applications, metagenomics is becoming an important approach for studying microbial communities in environments and/or the human body. Analyzing shotgun metagenomic data by assessing similarity to existing genome sequence databases is limited by the imcompleteness of these databases, especially for environments that have not been extensively studied. We conducted a systematic study on the application of sequence signature methods for the comparison of metagenomic samples, and found that, if dissimilarity measures are chosen properly, alignment-free methods based on sequence signatures can successfully reveal major group and gradient relationships among metagenomic samples from NGS reads without using reference databases or sequence assembly. These observations showed that analysis of sequence signatures provides an additional tool to evaluate patterns in microbial community data, and this tool is not subject to the biases in existing databases.

## Methods

### Simulated metagenomic datasets

We simulated four NGS metagenomic datasets using MetaSim [47]. In the first simulated datasets, the simulated communities consist of 5 bacterial species: *Acidobacterium capsulatum*, *Bacteroides fragilis*, *Nitrosospira multiformis*, *Proteus mirabilis* and *Sulfolobus islandicus* found in a soil bacterial community from a previous study [42]. Two types of sample relationships were simulated: group relationship where the species abundance levels of the samples belong to different groups and gradient relationship where the species abundance levels change continuously with some variables such as temperature or location.

In **Simulation 1**, we simulated 90 samples belonging to 3 groups (shown in Additional file 1: Figure S11). We began with a relative abundance vector of the 5 bacterial species (0.05,0.10,0.20,0.25,0.40), and each component was perturbed by multiplying the absolute value of a normally distributed variable with mean 1 and standard deviation equal to the value of the component itself. The relative abundance vector was renormalized to sum to 1. The original species relative abundance vector was perturbed and renormalized in this manner for three times independently, and three relative abundance vectors were obtained: (0.022, 0.058, 0.116, 0.507, 0.297), (0.042, 0.088, 0.281, 0.244, 0.345) and (0.046, 0.066, 0.042, 0.320, 0.526). We used these three vectors as the species abundance vectors for the three group centers and further generated three groups of species relative abundance vectors around them. For each group center relative abundance vector, we added to each component the absolute value of a Gaussian noise of mean zero and standard derivation equal to each component and renormalized components to sum to 1. Each relative abundance vector was randomized and renormalized 30 times, and a total of 90 relative abundance vectors were obtained. With these relative abundance vectors, we generated 90 metagenomic samples by mixing the 5 bacterial genomes at the abundance levels defined in the vectors and sampling short reads from the mixture genomes with MetaSim [47]. The sampling procedure mimics the next-generation sequencing by the Roche/454 platform with read length of 200nt.

In **Simulation 2**, we simulated 20 samples consisting of the same 5 bacterial species as in Simulation 1 and the relative abundance vector of the 5 species shifts along a gradient axis. Among the 5 bacterial species, we set the abundance level of one of them as constant, and let the abundance levels of the other 4 species vary following 4 functions across the sampling indexes. The four functions (shown in Additional file 1: Figure S12) have centers approximately around sample indexes 0, 5, 10 and 15, respectively. The abundances of all 4 species were first taken from these functions and were then normalized together with the species with constant abundance to sum to 1, forming the relative abundance vectors. Absolute values of Gaussian noises were added to each component of the abundance vector, with mean 0 and standard deviation equal to the value of each component. The vectors were renormalized after adding the noises. We generated 20 metagenomic samples by sampling the 5 bacterial genomes according to these relative abundance vectors using MetaSim [47] with read length of 200nt.

In **Simulations 3 and 4**, we considered more complex communities consisting of 113 microbial genomes from the collection of genomes provided by the FAMeS dataset [45,46]. The relative abundance vectors of the microbial genomes were generated from the power-law (Zipf’s) distribution:

(1) $$f\left(k;\alpha ,N\right)=\frac{1/k^{\alpha }}{\sum_{n}^{N}1/n^{\alpha }}\;,\;\text{with}\;\alpha \;=\;0.3,N=113,\;\text{and}\;k=1,\ldots N\text{.}$$

In **Simulation 3**, we generated 60 samples belonging to 3 groups with 20 samples in each group. For each group, we randomly ordered the 113 genomes and assigned the *i*-th genome with relative abundance *f (k;α,N)*. Then we added to each component the absolute value of a Gaussian noise with mean zero and standard derivation equal to each component and renormalized components to sum to 1. Each relative abundance vector was randomized and renormalized 20 times, and a total of 60 relative abundance vectors were obtained. Then we used these relative abundance vectors to simulate 60 metagenomic samples.

In **Simulation 4**, we generated 20 samples consisting of the same 113 genomes , and the relative abundance vector of the 113 genomes were generated by the power-law (Zipf’s) distribution as in the third simulation. In order to mimic the gradient model, the relative abundance vector shifts along a gradient axis of α from 0.275 to 0.75 by 0.025. Again, absolute values of Gaussian noises were added to each component of the 20 abundance vectors, with mean 0 and standard deviation equal to the value of that component. The vectors were renormalized after adding the noises. We generated 20 metagenomic samples according to these relative abundance vectors using MetaSim. In order to see the effect of sequencing platform, we generated short reads through both Roche/454 and Illumina/Solexa platforms by their default parameters in Simulations 3 and 4.

In all the simulations, we generated datasets at three sequencing depths: 1,000, 10,000 and 100,000 sequencing reads per sample. At each setting, we generated 100 duplicated datasets to simulate possible stochastic effects in real NGS data.

### Real metagenomic datasets

We analyzed three real shotgun metagenomic datasets published in recent years.

**The Mammalian Gut Dataset** includes 39 fecal microbiome samples from 33 mammalian species [31]. Shotgun sequencing of whole metagenome by the multiplex shotgun Roche/454 FLX pyrosequencing platform produced a total of 2,163,286 reads [mean = 55,469±28,724(SD) per sample] (Additional file 1: Table S1). The read length is 261±83(SD) nt. The animals in the dataset represent three diet types and varied digestive physiologies (hindgut-fermenting herbivores, *n* = 8; foregut-fermenting herbivores, *n* = 13; simple-gut carnivores, *n* = 7, and simple-gut omnivores, *n* = 11). As reported in previous studies with both targeted 16S rRNA sequencing and whole metagenome sequencing, the fecal microbiomes of herbivores and carnivores are associated with host diets and digestive physiologies [9]. Furthermore, bacterial compositions of omnivores are very diverse and cannot be distinguished from other groups of mammals [31]. Therefore, we studied this dataset in two steps. First, we excluded the omnivore samples and only studied the relationships among the groups of hindgut-fermenting herbivores, foregut-fermenting herbivores and simple-gut carnivores. Then we included the omnivore samples to study how the omnivore samples clustered with the other samples.

**The Global Ocean Dataset** includes 56 global ocean microbiome samples from 41 aquatic, largely marine locations across a multi-thousand kilometer transection from the North Atlantic through the Panama Canal and ending in the South Pacific [29]. Shotgun sequencing of whole metagenome by the AB3730xl platform produced 7,697,926 reads [mean = 137,463±145,307 (SD) per sample] (Additional file 1: Table S2). The read length is 1,032±58(SD) nt. In addition to different geographic locations, these samples also represent a range of habitat types, such as open ocean (*n* = 23), coastal (*n* = 19), and estuary (*n* = 3). As reported in previous studies, the diversity among samples shows significant separation between groups located in temperate regions and those located in tropical regions, although certain outliers can be present [29]. In our study, we first applied the sequence signature methods to the 23 open ocean samples and 19 coastal ocean samples separately to avoid possible interactive effects of sampling location and habitat types. Finally, the methods were applied to all 56 samples to obtain a panoramic view of the microbial communities.

**The Human Gut Dataset** includes 13 fecal microbiome metagenomic samples from healthy Japanese individuals, consisting of 7 adults, 2 weaned children, and 4 unweaned infants (Additional file 1: Table S4) [28]. Shotgun sequencing of the whole metagenome produced 1,041,617 reads [mean = 80,124±2,619(SD) per sample] with read length of 756±162(SD) nt. These samples were collected from 2 families (Family I: 2 adults and 2 weaned children; Family II: 2 adults and 1unweaned infant), 4 individual adults, and 2 individual infants. The previous study revealed intriguing differences in microbiomes between adults and unweaned infants and a high functional uniformity in adults and weaned children [28]. In our study, sequence signature methods were applied to detect the dissimilarity between the groups of samples.

### The *k*-tuple count vectors and dissimilarity measures

The comparison of metagenomic samples using sequence signatures arises from the need for the comparison of two microbial community samples without having to do alignment with reference genomes, which are often incomplete or unavailable. For every metagenomic sample, we can obtain its *k*-tuple count vector as the sequence signature. Based on these vectors, we can use a dissimilarity measure to evaluate the difference between two samples. We analyze the relationships among multiple samples using the dissimilarity matrix composed of the dissimilarities between all pairs of samples.

The first step is to count the number of occurrences of each *k*-tuple. Reads in NGS data can come from either the forward strand or the reverse strand of a genome. Because we do not know which strand a read comes from, we consider both a read and its complement to take both strands into consideration. That is, we supplement the observed reads by their complements to form the read set on which the number of *k*-tuple occurrences are counted. For genome or metagenome data, we have a finite alphabet set *S* = {" A ", " C ", " G ", " T "} and the set of all possible k-tuples *W* = {*w* ∈ *S*^*k*^}. The numbers of occurrences of all the 4^k^ tuples of length *k* in all reads of a metagenome sample form the *k*-tuple count vector of the sample.

In this paper, we studied a collection of dissimilarity measures between *k*-tuple count vectors, including three *d*_*2*_-type dissimilarity measures *d*_*2*_, *d*_2_^*S*^ , and *d*_2_^*^[34]. For the calculation of *d*_2_^*S*^ and *d*_2_^*^, we need to centralize the real count of a tuple by deducting its expected number of occurrences under certain models for the underlying background genome sequences. We estimated the background genome sequences using Markov models of orders 0, 1, 2, and 3, respectively. In addition to the *d*_*2*_-type dissimilarities, we also studied the Euclidean, Manhattan, and Chebyshev distance measures for the *k*-tuple frequency vectors. We also studied a dissimilarity measure developed from Dr. Hao’s group (*Hao*) [41] and this measure uses a Markov model of order *k*-2 for the background sequences. We next describe these measures in detail.

Let $c_{X}=\left(c_{X,1},c_{X,2},\ldots ,c_{X,4^{k}}\right)$ and $c_{Y}=\left(c_{Y,1},c_{Y,2},\ldots ,c_{Y,4^{k}}\right)$ be the *k*-tuple count vectors of two metagenomic samples X and Y, respectively. The *d*_*2*_ dissimilarity measure [34] derived from the well-known D_2_ statistic [48] is

(2) $$\begin{array}{c} D_{2}\left(c_{X},c_{Y}\right)=\sum_{i=1}^{4^{k}}{c_{X}}_{,i}{c_{Y}}_{,i}\\ d_{2}\left(c_{X},c_{Y}\right)=\frac{1}{2}\left(1-\frac{D_{2}\left(c_{X},c_{Y}\right)}{\sqrt{\sum_{i=1}^{4^{k}}c_{X,i}^{2}}\sqrt{\sum_{i=1}^{4^{k}}c_{Y,i}^{2}}}\right) \end{array}$$

Dissimilarity measures *d*_2_^*S*^ and *d*_2_^*^[34] derived from two variants of D_2_ termed *D*_2_^*S*^ and *D*_2_^*^[49], were also studied. To define *d*_2_^*S*^ and *d*_2_^*^, we first introduce the centralized count variables by

${\tilde{c}}_{X,i}=c_{X,i}-n_{X}p_{X,i}$ and ${\tilde{c}}_{Y,i}=c_{Y,i}-n_{Y}p_{Y,i}$

where *p*_·,*i*_ is the probability of *i* th *k*-tuple under the probability model (Markov model of order *r*=0,1,2,3) for the background sequences and $n_{\cdot }=\sum_{i=1}^{4^{k}}c_{\cdot ,i}$ is the total count of *k*-tuples. Then we have

(3) $$\begin{array}{c} D_{2}^{S}\left({\tilde{c}}_{X},{\tilde{c}}_{Y}\right)=\sum_{i=1}^{4^{k}}\frac{{\tilde{c}}_{X,i}{\tilde{c}}_{Y,i}}{\sqrt{{\tilde{c}}_{X,i}^{2}+{\tilde{c}}_{Y,i}^{2}}}\\ d_{2}^{S}\left({\tilde{c}}_{X},{\tilde{c}}_{Y}\right)=\frac{1}{2}\left(1-\frac{D_{2}^{S}\left({\tilde{c}}_{X},{\tilde{c}}_{Y}\right)}{\sqrt{\sum_{i=1}^{4^{k}}\frac{{\tilde{c}}_{X,i}^{2}}{\sqrt{{\tilde{c}}_{X,i}^{2}+{\tilde{c}}_{Y,i}^{2}}}}\sqrt{\sum_{i=1}^{4^{k}}\frac{{\tilde{c}}_{Y,i}^{2}}{\sqrt{{\tilde{c}}_{X,i}^{2}+{\tilde{c}}_{Y,i}^{2}}}}}\right) \end{array}$$

and

(4) $$\begin{array}{c} D_{2}^{*}\left({\tilde{c}}_{X},{\tilde{c}}_{Y}\right)=\sum_{i=1}^{4^{k}}\frac{{\tilde{c}}_{X,i}{\tilde{c}}_{Y,i}}{\sqrt{n_{X}p_{X,i}}\sqrt{n_{Y}p_{Y,i}}}\\ d_{2}^{*}\left({\tilde{c}}_{X},{\tilde{c}}_{Y}\right)=\frac{1}{2}\left(1-\frac{D_{2}^{*}\left({\tilde{c}}_{X},{\tilde{c}}_{Y}\right)}{\sqrt{\sum_{i=1}^{4^{k}}\frac{{\tilde{c}}_{X,i}^{2}}{n_{X}p_{X,i}}}\sqrt{\sum_{i=1}^{4^{k}}\frac{{\tilde{c}}_{X,i}^{2}}{n_{Y}p_{Y,i}}}}\right) \end{array}$$

The ranges of both *d*_2_^*S*^ and *d*_2_^*^ are between 0 and 1. When the two samples are the same, both *d*_2_^*S*^ and *d*_2_^*^ are 0. When the two samples are completely independent, the expected values of *D*_2_^*S*^ and *D*_2_^*^ are 0, and thus, the expected value of both *d*_2_^*S*^ and *d*_2_^*^ is 0.5. When the enriched tuples in the two samples complement each other, the value of both *d*_2_^*S*^ and *d*_2_^*^ is close to 0. Therefore, these measures can be used to measure the dissimilarity between the samples.

In addition to the newly developed *d*_*2*_-type dissimilarity measures, we also studied the standard *l*_*p*_-norm distance measures for *k*-tuple frequency vectors. We first normalized *k*-tuple count vectors to *k*-tuple frequency vectors whose components sum to 1,$f_{X}=\frac{c_{X}}{n_{X}}$ and $f_{Y}=\frac{c_{Y}}{n_{Y}}$

where $n_{X}=\sum_{i=1}^{4^{k}}c_{X,i}$ and $n_{Y}=\sum_{i=1}^{4^{k}}c_{Y,i}$. Three classical *l*_*p*_-norm distances including Manhattan (*l*_*1*_-norm), Euclidean (*l*_*2*_-norm) and Chebyshev (*l∞-*norm), abbreviated as *Ma*, *Eu* and *Ch*, respectively, are defined to compare *k*-tuple frequency vectors.

(5) $$\ell_{p}\left(f_{X},f_{Y}\right)={\left(\sum_{i=1}^{4^{k}}{\left|f_{X,i}-f_{Y,i}\right|}^{p}\right)}^{1/p}$$

(6) $$\mathrm{Ma}\left(f_{X},f_{Y}\right)=\ell_{1}\left(f_{X},f_{Y}\right)=\sum_{i=1}^{4^{k}}\left|f_{X,i}-f_{Y,i}\right|$$

(7) $$\mathrm{Eu}\left(f_{X},f_{Y}\right)=\ell_{2}\left(f_{X},f_{Y}\right)={\left(\sum_{i=1}^{4^{k}}{\left|f_{X,i}-f_{Y,i}\right|}^{2}\right)}^{1/2}$$

(8) $$\mathrm{Ch}\left(f_{X},f_{Y}\right)=\ell_{\infty }\left(f_{X},f_{Y}\right)=\max_{1\leq i\leq 4^{k}}\left|f_{X,i}-f_{Y,i}\right|$$

A dissimilarity measure developed by Qi et al. [41] from Dr. Hao’s group is also studied. We use the corresponding author’s last name *Hao* as the short form of this measure to simplify the notation. *Hao* is based on the relative difference between the actual *k*-tuple frequencies of each word with its expectation under a Markov model of order *k*–2.

(9) $$\text{Hao}=\frac{\sum_{i=1}^{4^{k}}\left(\frac{f_{X,i}}{E\left[f_{X,i}|M_{k-2}\right]}-1\right)\left(\frac{f_{Y,i}}{E\left[f_{Y,i}|M_{k-2}\right]},-,1\right)}{\sqrt{\sum_{i=1}^{4^{k}}{\left(\frac{f_{X,i}}{E\left[f_{X,i}|M_{k-2}\right]}-1\right)}^{2}}\sqrt{\sum_{i=1}^{4^{k}}{\left(\frac{f_{Y,i}}{E\left[f_{Y,i}|M_{k-2}\right]}-1\right)}^{2}}}$$

A new dissimilarity measure developed by Willner *et al*. [38] was also studied in this paper. The relative abundance odds ratio measure of dinucleotide was defined as $\rho_{\mathrm{XY}}^{*}=\frac{f_{\mathrm{XY}}}{f_{X}f_{Y}}$. The relative abundance odds ratio for tri-nucleotides was defined as $\gamma_{\text{XYZ}}^{*}=\frac{f_{\text{XYZ}}f_{X}f_{Y}f_{Z}}{f_{\mathrm{XY}}f_{\mathrm{YZ}}f_{\mathrm{XNZ}}}$, and for tetra-nucleotides was defined as $\tau_{\text{XYZW}}^{*}=\frac{f_{\text{XYZW}}f_{\mathrm{XY}}f_{\text{XNZ}}f_{\text{XNMW}}f_{\mathrm{YZ}}f_{\text{YNW}}f_{\mathrm{ZW}}}{f_{\text{XYZ}}f_{\text{XYNW}}f_{\text{YZW}}f_{X}f_{Y}f_{Z}f_{W}}$, where *N* and *M* represent any nucleotide [32].

The new dissimilarity measures, termed *Willner*, based on the relative abundance odds ratios for di-, tri-, and tetra-nucleotides were defined as

(10) $$\delta_{2}\left(f,g\right)=\frac{1}{4^{2}}\sum_{\mathrm{XY}}\left|\rho_{\mathrm{XY}}\left(f\right)-\rho_{\mathrm{XY}}\left(g\right)\right|$$

(11) $$\delta_{3}\left(f,g\right)=\frac{1}{4^{3}}\sum_{\text{XYZ}}\left|\gamma_{\text{XYZ}}\left(f\right)-\gamma_{\text{XYZ}}\left(g\right)\right|$$

(12) $$\delta_{4}\left(f,g\right)=\frac{1}{4^{4}}\sum_{\text{XYZW}}\left|\tau_{\text{XYZW}}\left(f\right)-\tau_{\text{XYZW}}\left(g\right)\right|$$

For *d*_2_^*S*^ and *d*_2_^*^ and the *Hao* dissimilarity measures described above, the expected number of occurrences of each tuple needs to be calculated. To do this, we need to assume a model for the background sequence. In this study, we used Markov models of different orders (0, 1, 2, and 3 for *d*_2_^*S*^ and *d*_2_^*^ , and *k*–2 for *Hao*) to model the background genome sequences. Markov model of order 0 corresponds to the independent identically distributed (i.i.d.) model where genome sequences are generated as a series of independent and identically distributed random characters. Thus, for a *k*-tuple *w* = *w*_1_*w*_2_ … *w*_*k*_, the expected frequency under the Markov model of order 0 (M_0_) is also the probability of *w* occurring under M_0_:

(13) $$E\left[f\left(w_{1}w_{2}\ldots w_{k}\right)|M_{0}\right]=p_{w}=p\left(w_{1}w_{2}\ldots w_{k}\right)=\prod_{j=1}^{k}p\left(w_{j}\right)$$

where *p(w*_*j*_*)* is the probability of *w*_*j*_ in all the reads of a metagenomic sample.

Under Markov model (*M*_*r*_) of order *1≤ r ≤ k - 2* the expected frequency is

(14) $$E\left[f\left(w_{1}w_{2}\ldots w_{k}\right)|M_{r}\right]=p\left(w_{1}w_{2}\ldots w_{r}\right)\prod_{j=2}^{k-r}p\left(w_{j+r}|w_{j}w_{j+1}\ldots w_{j+r-1}\right)$$

where *p(w*_*1*_*w*_*2*_*…w*_*r*_*)* is the initial distribution of consecutive states *w*_*1*_*w*_*2*_*…w*_*r*_ in all the reads of a metagenomic sample, and *p*(*w*_*j* + *r*_|*w*_*j*_*w*_*j* + 1_ … *w*_*j* + *r* − 1_) is the transition probability going from consecutive states *w*_*j*_*w*_*j* + 1_ … *w*_*j* + *r* − 1_ to state *w*_*j* + *r*_.

### Beta-diversity analysis and evaluation methods

Detection of group relationships among microbial samples and the identification of external gradients driving shifts in microbial community structure are two major types of analysis tasks in microbial community comparison. Therefore, we evaluated the performance of the *k*-tuple dissimilarity measures in metagenomic comparison by assessing how well a method detects the known group relationships or identifies known environmental gradient.

For clustering analysis, we detect group relationships among the microbial samples by applying the UPGMA (unweighted pair-group method with arithmetic means) algorithm [43] based on the between-samples dissimilarity matrices. It is a hierarchical clustering algorithm based on pair-wise dissimilarity matrix of multiple samples, using average-linkage for measuring the dissimilarity of two clusters. We used the *phangorn*[50] package in R for this algorithm. We used the TreeClimber package [25], a tool to compare clustering of microbial communities, to evaluate the resulting clustering trees by the parsimony test. The parsimony score measures how far away the clustering tree is from the true classification of the samples. If samples in the same group are clustered together in a clustering tree, the parsimony score is *c*-1 where *c* is the number of groups. As the parsimony score increases, the clustering tree becomes increasingly different from the true grouping of the samples. Monte Carlo *p-*value is used to see whether the observed parsimony score is statistically significant. Here, samples were randomly grouped to generate clustering trees 1,000 times, and the *p-*value was approximated by the fraction of times that the parsimony score for the randomized trees is smaller than or equal to the parsimony score of the observed tree.

For the study of gradient relationships among the samples, the shift of microbial samples is visualized by PCoA (Principal Coordinates Analysis) [44], which is a multi-dimensional scaling (MDS) method that converts between-sample dissimilarity matrix into two-dimensional (or three-dimensional) ordinates of samples and arranges the samples in ordinate space. We used the MASS [51] package in R for PCoA. Then, the influence of environmental gradient(s) on microbial communities can be tested by calculating correlation, such as Pearson correlation coefficient (PCC), between PCoA axis and gradient axis. In this way, the performance of the between-sample dissimilarity measures given by sequence signature methods can be evaluated if the gradient driving microbial communities is known.

## Competing interest

All authors declare that they have no competing interest.

## Authors’ contributions

FS and XZ initiated the project and designed the study. BJ and JR wrote the programs for *k*-tuple counting. KS wrote the programs for background probability estimation and dissimilarity matrix calculation. JR collected the real datasets and generated the simulation datasets. BJ and KS analyzed the data. FS and MD guided the analysis of dissimilarity measures. BJ drafted the manuscript under the supervision of FS and XZ. All authors contributed to manuscript revision and agreed to the final manuscript.

## Supplementary Material

Additional file 1 Supplementary Materials for “Comparison of Metagenomic Samples Using Sequence Signatures”. Click here for file

## References

[B1] Lozupone C Lladser ME Knights D Stombaugh J Knight R UniFrac: an effective distance metric for microbial community comparison ISME J 2007 5 169 172 20827291 10.1038/ismej.2010.133 PMC3105689

[B2] Highton R The relationship between the number of loci and the statistical support for the topology of UPGMA trees obtained from genetic distance data Mol Phylogenet Evol 1993 2 337 343 10.1006/mpev.1993.1033 8049782

[B3] Krzanowski WJ Principles of multivariate analysis: a user’s perspective 2000 Oxford: Oxford University Press

[B4] Zhou J Xia B Treves DS Wu LY Marsh TL O’Neill RV Palumbo AV Tiedje JM Spatial and resource factors influencing high microbial diversity in soil Appl Environ Microbiol 2002 68 326 334 10.1128/AEM.68.1.326-334.2002 11772642 PMC126564

[B5] Roesch LFW Fulthorpe RR Riva A Casella G Hadwin AKM Kent AD Daroub SH Camargo FAO Farmerie WG Triplett EW Pyrosequencing enumerates and contrasts soil microbial diversity ISME J 2007 1 283 290 18043639 10.1038/ismej.2007.53 PMC2970868

[B6] Nakagawa T Ishibashi JI Maruyama A Yamanaka T Morimoto Y Kimura H Urabe T Fukui M Analysis of dissimilatory sulfite reductase and 16S rRNA gene fragments from deep-sea hydrothermal sites of the Suiyo Seamount, Izu-Bonin Arc. Western Pacific Appl Environ Microbiol 2004 70 393 403 10.1128/AEM.70.1.393-403.2004 14711668 PMC321305

[B7] Sogin ML Morrison HG Huber JA Welch DM Huse SM Neal PR Arrieta JM Herndl GJ Microbial diversity in the deep sea and the underexplored “rare biosphere” Proc Natl Acad Sci USA 2006 103 12115 12120 10.1073/pnas.0605127103 16880384 PMC1524930

[B8] Huber JA Welch DBM Morrison HG Huse SM Neal PR Butterfield DA Sogin ML Microbial population structures in the deep marine biosphere Science 2007 318 97 100 10.1126/science.1146689 17916733

[B9] Ley RE Hamady M Lozupone C Turnbaugh PJ Ramey RR Bircher JS Schlegel ML Tucker TA Schrenzel MD Knight R Gordon JI Evolution of mammals and their gut microbes Science 2008 320 1647 1651 10.1126/science.1155725 18497261 PMC2649005

[B10] Fierer N Hamady M Lauber CL Knight R The influence of sex, handedness and washing on the diversity of hand surface bacteria Proc Natl Acad Sci USA 2008 105 17994 17999 10.1073/pnas.0807920105 19004758 PMC2584711

[B11] Costello EK Lauber CL Hamady M Fierer N Gordon JI Knight RL Bacterial community variation in human body habitats across space and time Science 2009 326 1694 1697 10.1126/science.1177486 19892944 PMC3602444

[B12] Grice EA Kong HH Conlan S Deming CB Davis J Young AC Bouffard GG Blakesley RW Murray PR Green ED Turner ML Segre JA Topographical and temporal diversity of the human skin microbiome Science 2009 324 1190 1192 10.1126/science.1171700 19478181 PMC2805064

[B13] Andersson AF Lindberg M Jakobsson H Backhed F Nyren P Engstrand L Comparative analysis of human gut microbiota by barcoded pyrosequencing PLoS One 2008 3 e2836 10.1371/journal.pone.0002836 18665274 PMC2475661

[B14] Turnbaugh PJ Hamady M Yatsunenko T Cantarel BL Duncan A Ley RE Sogin ML Jones WJ Roe BA Affourtit JP Egholm M Henrissat B Heath AC Knight R Gordon JI A core gut microbiome in obese and lean twins Nature 2009 457 480 484 10.1038/nature07540 19043404 PMC2677729

[B15] Turnbaugh PJ Quince C Faith JJ McHardy AC Yatsunenko T Niazi F Affourtit J Egholm M Henrissat B Knight R Gordon JI Organismal, genetic, and transcriptional variation in the deeply sequenced gut microbiomes of identical twins Proc Natl Acad Sci USA 2010 107 7503 7508 10.1073/pnas.1002355107 20363958 PMC2867707

[B16] Keijser BJF Zaura E Huse SM der VossenJ V Schuren FHJ Montijn RC Cate JMT Crielaard W Pyrosequencing analysis of the oral microflora of healthy adults J Dent Res 2008 87 1016 1020 10.1177/154405910808701104 18946007

[B17] Nasidze I Li J Quinque D Tang K Stoneking M Global diversity in the human salivary microbiome Genome Res 2009 19 636 643 10.1101/gr.084616.108 19251737 PMC2665782

[B18] Lazarevic V Whiteson K Huse S Hernandez D Farinelli L Østerås M Schrenzel J François P Metagenomic study of the oral microbiota by Illumina high-throughput sequencing J Microbiol Methods 2009 79 266 271 10.1016/j.mimet.2009.09.012 19796657 PMC3568755

[B19] Kuczynski J Liu Z Lozupone C McDonald D Fierer N Knight R Microbial community resemblance methods differ in their ability to detect biologically relevant patterns Nat Methods 2010 7 10 813 9 10.1038/nmeth.1499 20818378 PMC2948603

[B20] Lozupone CA Knight R UniFrac: a new phylogenetic method for comparing microbial samples Appl Environ Microbiol 2005 71 8228 8235 10.1128/AEM.71.12.8228-8235.2005 16332807 PMC1317376

[B21] Lozupone C Hamady M Knight R UniFrac: an online tool for comparing microbial community diversity in a phylogenetic context BMC Bioinformatics 2006 7 371 10.1186/1471-2105-7-371 16893466 PMC1564154

[B22] Hamady M Lozupone C Knight R Fast UniFrac: facilitating high-throughput phylogenetic analyses of microbial communities including analysis of pyrosequencing and PhyloChip data ISME J 2010 4 17 27 10.1038/ismej.2009.97 19710709 PMC2797552

[B23] Chang Q Luan Y Sun FZ Variance adjusted weighted UniFrac: a powerful beta diversity measure for comparing communities based on phylogeny BMC Bioinformatics 2011 12 118 10.1186/1471-2105-12-118 21518444 PMC3108311

[B24] Schloss PD Handelsman J Introducing DOTUR, a computer program for defining operational taxonomic units and estimating species richness Appl Environ Microbiol 2005 71 1501 1506 10.1128/AEM.71.3.1501-1506.2005 15746353 PMC1065144

[B25] Schloss PD Handelsman J Introducing TreeClimber, a test to compare microbial community structures Appl Environ Microbiol 2006 72 2379 2384 10.1128/AEM.72.4.2379-2384.2006 16597933 PMC1449046

[B26] Huson DH Auch AF Qi J Schuster SC MEGAN analysis of metagenomic data Genome Res 2007 17 377 386 10.1101/gr.5969107 17255551 PMC1800929

[B27] Gill SR Pop M DeBoy RT Eckburg PB Turnbaugh PJ Samuel BS Gordon JI Relman DA Fraser-Liggett CM Nelson KE Metagenomic analysis of the human distal gut microbiome Science 2006 312 1355 1359 10.1126/science.1124234 16741115 PMC3027896

[B28] Kurokawa K Itoh T Kuwahara T Oshima K Toh H Toyoda A Takami H Morita H Sharma VK Srivastava TP Taylor TD Noguchi H Mori H Ogura Y Ehrlich DS Itoh K Takagi T Sakaki Y Hayashi T Hattori M Comparative metagenomics revealed commonly enriched gene sets in human gut microbiomes DNA Res 2007 14 169 181 10.1093/dnares/dsm018 17916580 PMC2533590

[B29] Rusch DB Halpern AL Sutton G Heidelberg KB Williamson S Yooseph S Wu DY Eisen JA Hoffman JM Remington K Beeson K Tran B Smith H Baden-Tillson H Stewart C Thorpe J Freeman J Andrews-Pfannkoch C Venter JE Li K Kravitz S Heidelberg JF Utterback T Rogers YH Falcon LI Souza V Bonilla-Rosso G Eguiarte LE Karl DM Sathyendranath S The Sorcerer II global ocean sampling expedition: northwest Atlantic through eastern tropical pacific PLoS Biol 2007 5 398 431 10.1371/journal.pbio.0050077 PMC1821060 17355176

[B30] Qin J Li R Raes J Arumugam M Burgdorf KS Manichanh C Nielsen T Pons N Levenez F Yamada T Mende DR Li J Xu J Li S Li D Cao J Wang B Liang H Zheng H Xie Y Tap J Lepage P Bertalan M Batto JM Hansen T Denis Le P Linneberg A Nielsen HB Pelletier E Renault P A human gut microbial gene catalogue established by metagenomic sequencing Nature 2010 464 59 65 10.1038/nature08821 20203603 PMC3779803

[B31] Muegge BD Kuczynski J Knights D Clemente JC Gonzalez A Fontana L Henrissat B Knight R Gordon JI Diet drives convergence in gut microbiome functions across mammalian phylogeny and within humans Science 2011 332 970 973 10.1126/science.1198719 21596990 PMC3303602

[B32] Karlin S Mrazek J Campbell AM Compositional biases of bacterial genomes and evolutionary implications J Bacteriol 1997 179 3899 3913 9190805 10.1128/jb.179.12.3899-3913.1997 PMC179198

[B33] Pride DT Meinersmann RJ Wassenaar TM Blaser MJ Evolutionary implications of microbial genome tetranucleotide frequency biases Genome Res 2003 13 145 158 10.1101/gr.335003 12566393 PMC420360

[B34] Song K Ren J Zhai Z Liu X Deng M Sun FZ Alignment-Free Sequence Comparison Based on Next Generation Sequencing Reads Res Comput Mol Biol 2012 7262 272 285 10.1007/978-3-642-29627-7_29 PMC3581251 23383994

[B35] Dalevi D Dubhashi D Hermansson M Bayesian classifiers for detecting HGT using fixed and variable order Markov models of genomic signatures Bioinformatics 2006 22 517 522 10.1093/bioinformatics/btk029 16403797

[B36] Teeling H Meyerdierks A Bauer M Amann R Glöckner FO Application of tetra-nucleotide frequencies for the assignment of genomic fragments Environ Microbiol 2004 6 938 947 10.1111/j.1462-2920.2004.00624.x 15305919

[B37] Dick GJ Andersson AF Baker BJ Simmons SL Thomas BC Yelton AP Banfield JF Community-wide analysis of microbial genome sequence signatures Genome Biol 2009 10 R85 10.1186/gb-2009-10-8-r85 19698104 PMC2745766

[B38] Willner D Thurber RV Rohwer F Metagenomic signatures of 86 microbial and viral metagenomes Environ Microbiol 2009 11 7 1752 1766 10.1111/j.1462-2920.2009.01901.x 19302541

[B39] Ghosh TS Mohammed MH Rajasingh H Chadaram S Mande SS HabiSign: a novel approach for comparison of metagenomes and rapid identification of habitat-specific sequences BMC Bioinformatics 2011 12 Suppl 13 59 22373355 10.1186/1471-2105-12-S13-S9 PMC3278849

[B40] Maillet N Lemaitre C Chikhi R Lavenier D Peterlongo P Compareads: comparing huge metagenomic experiments, presented at RECOMB Comparative Genomics 2012 2012 Brazil: Niteroi 10.1186/1471-2105-13-S19-S10 PMC3526429 23282463

[B41] Qi J Wang B Hao BL Whole proteome prokaryote phylogeny without sequence alignment: a k-string composition approach J Mol Evol 2004 58 1 11 10.1007/s00239-003-2493-7 14743310

[B42] Rousk J Baath E Brookes PC Lauber CL Lozupone C Caporaso JG Knight R Fierer N Soil bacterial and fungal communities across a pH gradient in an arable soil ISME J 2010 4 1340 1351 10.1038/ismej.2010.58 20445636

[B43] Murtagh F Complexities of hierarchic clustering algorithms: the state of the art Comput Stat Q 1984 1 101 113

[B44] Anderson MJ PCO: a FORTRAN computer program for principal coordinate analysis 2003 New Zealand: Department of Statistics, University of Auckland

[B45] Mavromatis K Ivanova N Barry K Shapiro H Goltsman E McHardy AC Rigoutsos I Salamov A Korzeniewski F Land M Use of simulated data sets to evaluate the fidelity of metagenomic processing methods Nat Methods 2007 4 495 500 10.1038/nmeth1043 17468765

[B46] Xia LC Cram JA Chen T Fuhrman JA Sun FZ Accurate genome relative abundance estimation based on shotgun metagenomic reads PLoS One 2011 6 12 e27992 10.1371/journal.pone.0027992 22162995 PMC3232206

[B47] Richter DC Ott F Auch AF Schmid R Huson DH MetaSim: A sequencing simulator for genomics and metagenomics PLoS One 2008 3 e3373 10.1371/journal.pone.0003373 18841204 PMC2556396

[B48] Blaisdell BE A measure of the similarity of sets of sequences not requiring sequence alignment Proc Natl Acad Sci USA 1986 83 5155 5159 10.1073/pnas.83.14.5155 3460087 PMC323909

[B49] Reinert G Chew D Sun FZ Waterman MS Alignment-free sequence comparison (I): statistics and power J Comput Biol 2009 12 1615 1634 20001252 10.1089/cmb.2009.0198 PMC2818754

[B50] Schliep KP Phangorn: phylogenetic analysis in R Bioinformatics 2011 27 4 592 593 10.1093/bioinformatics/btq706 21169378 PMC3035803

[B51] Venables WN Ripley BD Modern Applied Statistics with S 2002 New York: Springer

