# Supplementary material for: Comparison of metagenomic samples using sequence signatures
Source: BMC Genomics. 2012 Dec 27;13:730. doi: 10.1186/1471-2164-13-730 (PMC3549735; doi:10.1186/1471-2164-13-730)
Supplement: Additional file 1 — Supplementary Materials for “Comparison of Metagenomic Samples Using Sequence Signatures”. [file 1471-2164-13-730-S1.pdf]

Supplementary Materials for

"Comparison of Metagenomic Samples Using Sequence Signatures"

Bai Jiang, Kai Song, Jie Ren, Minghua Deng, Fengzhu Sun, Xuegong Zhang

Figure S1 – The effect of the order of Markov model on the performance of d_2^* (upper panel) and d_2^S (lower panel) at different sequencing depths to recover group relationship of metagenomic samples.

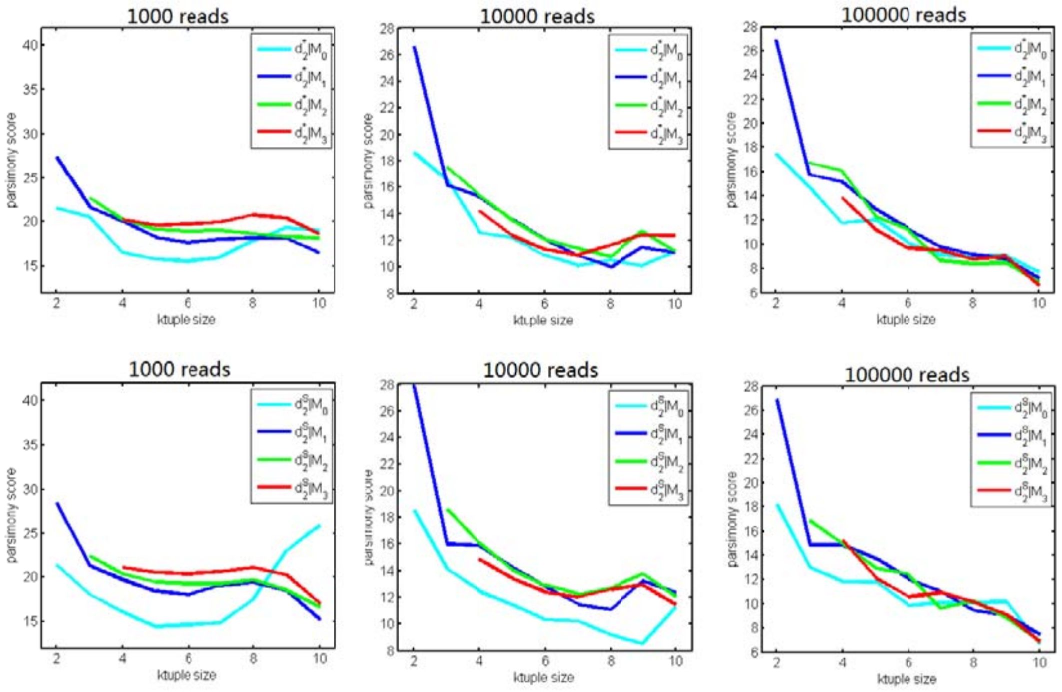

Figure S2 – The effects of sequencing depth and tuple size on the mean (a) and standard deviation (b) of the parsimony scores from 100 repeated simulations with dissimilarity measure $d_2^S|\mathrm{M}_0$ in Simulation 1.

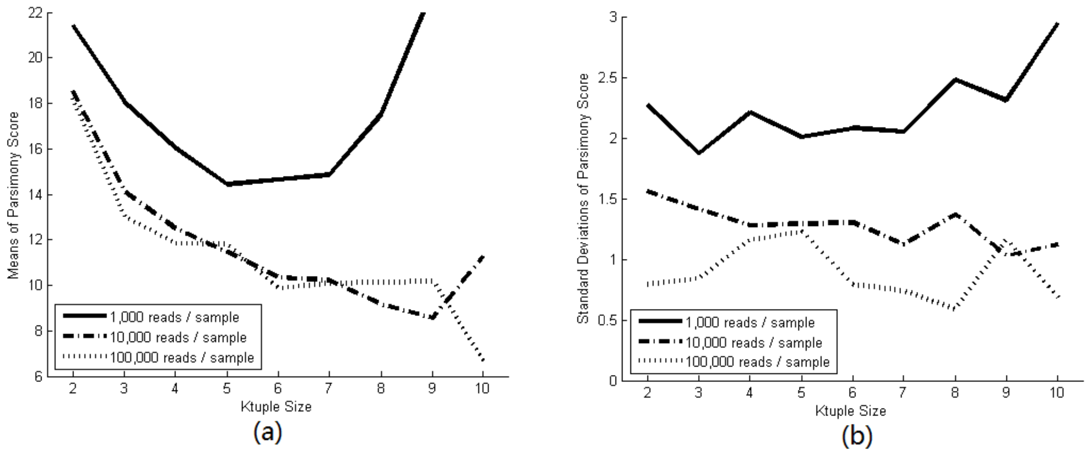

Figure S3 – The effect of the order of Markov model on the performance of d_2^* (upper panel) and d_2^S (lower panel) at different sequencing depths to recover gradient relationship of metagenomic samples. The order of the Markov model has little effect on the performance of d_2^* and d_2^S.

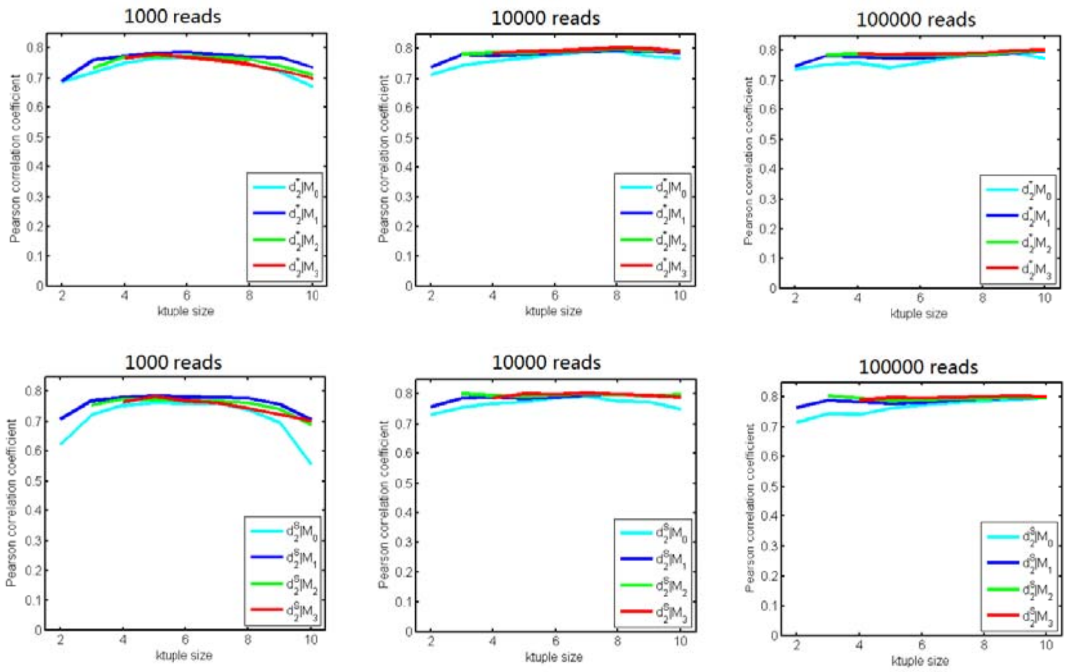

Figure S4 – The effects of sequencing depth and tuple size on the mean (a) and standard deviation (b) of the PCCs from 100 repeated simulations between the first principal coordinate (PC1) and the gradient with dissimilarity measure $d_2^S|\mathrm{M}_0$ in Simulation 2.

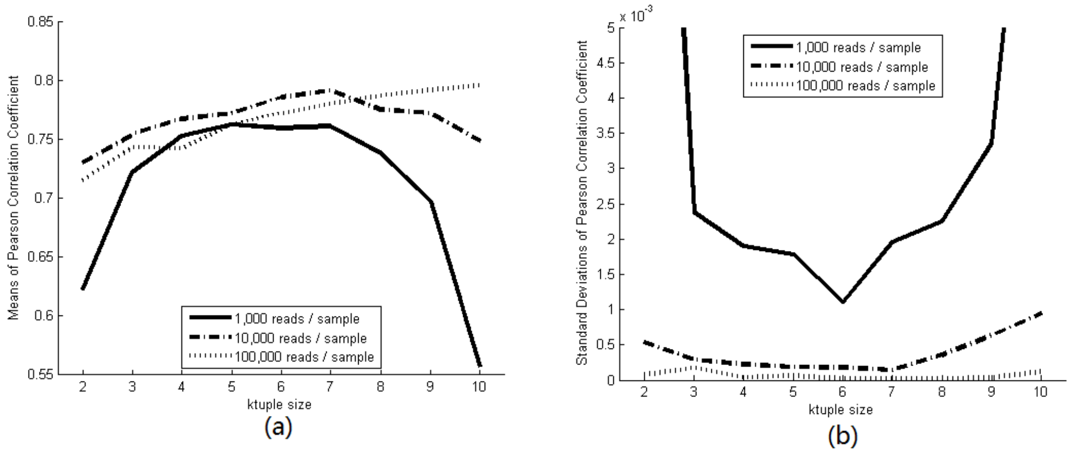

Figure S5 – (a) The average parsimony scores of clustering trees obtained in Simulation 3 using the Roche/454 platform with different tuple sizes, dissimilarity measures and sequencing depths of 1,000 , 10,000 and 100,000 reads per sample. (b) The effect of the order of Markov model on the performance of d_2^S and d_2^* at the sequencing depth of 10,000 reads per sample.

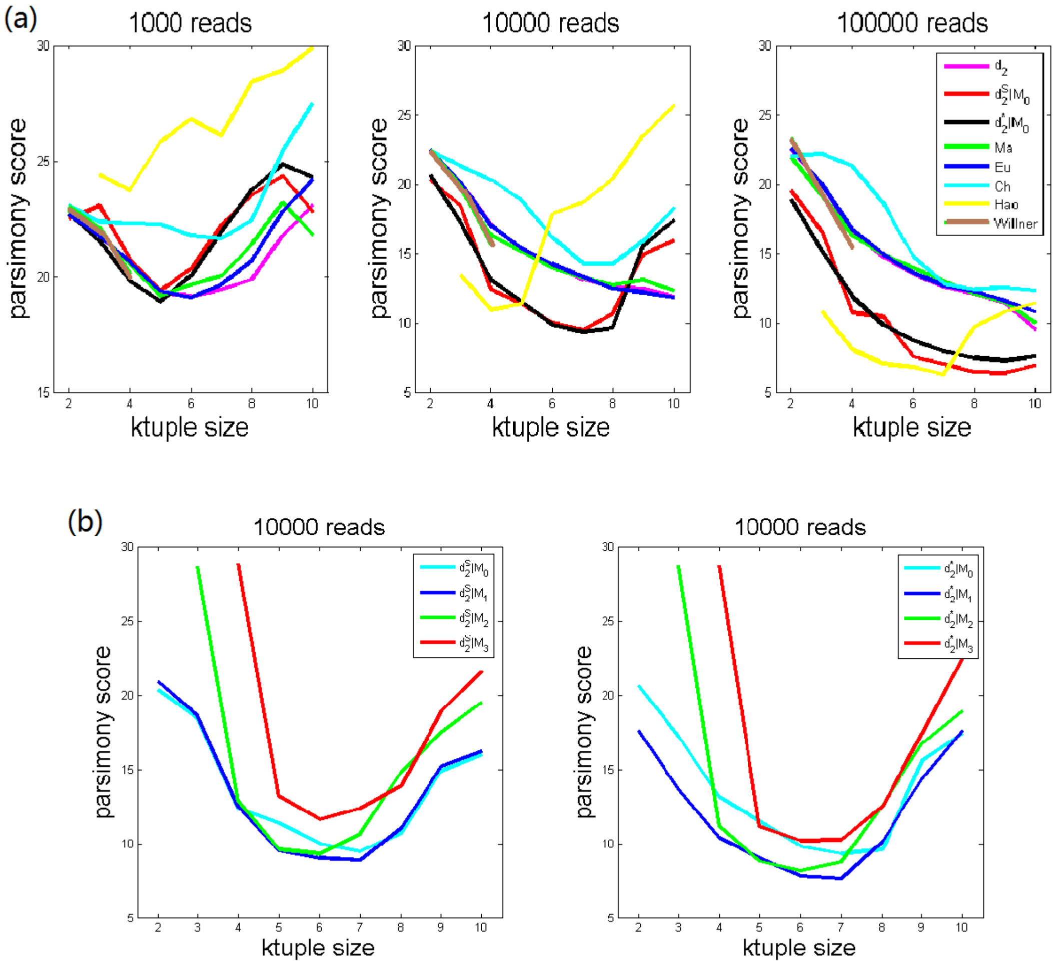

Figure S6 – The average PCC between PC1 and the gradient in Simulation 4 using the Roche/454 platform for different dissimilarity measures, tuple sizes, and sequencing depths of (a) 1,000 , 10,000 and 100,000 reads per sample. The dissimilarity measure d_2^S and d_2^* outperform others in recovering the gradient relationship among the samples. (b) The effect of the order of Markov model on the performance of d_2^S and d_2^* at the sequencing depth of 10,000 reads per sample.

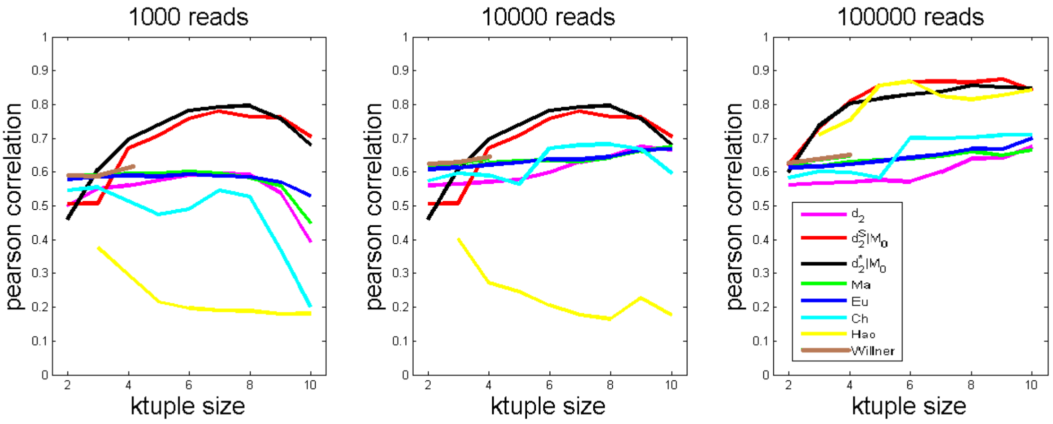

(a)

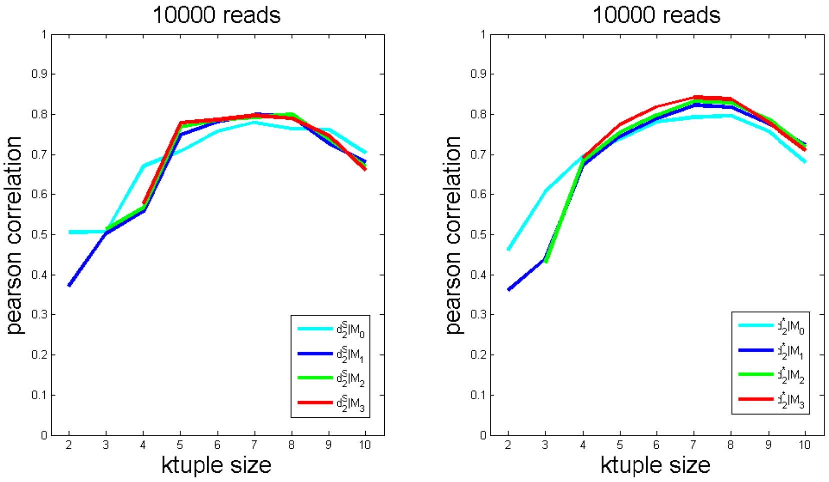

(b)

Figure S7- The relative performance of different dissimilarity measures for sequence data generated using the Illumina/Solexa platform at sequencing depth of 10,000 reads per sample. (a) The average parsimony scores of clustering trees obtained in Simulation 3 and (b) the average PCCs of PC1 and the gradient in Simulation 4 with different dissimilarity measures and different tuple sizes.

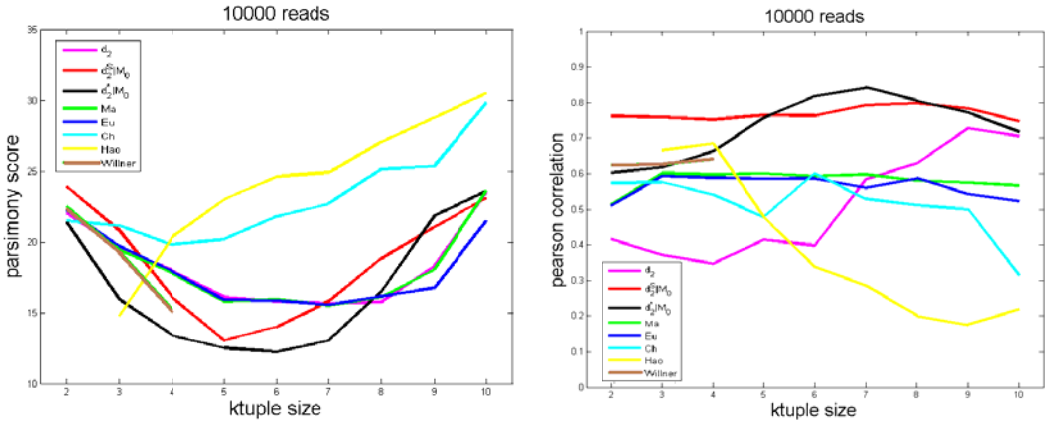

(a) (b)

Figure S8 – Ordination analysis of the mammalian gut samples based on 5-tuples and $d_2^S|M_0$.
Foregut-fermenting herbivore samples (green up-triangle), hindgut-fermenting herbivore samples (yellow down-triangle), simple-gut carnivore samples (red circle) and simple-gut omnivore samples (blue square). (a) Excluding omnivorous samples. Four pairs of samples from the same species are annotated with sample ID. (b) Including omnivorous samples. Three bear samples are annotated with sample ID.

(a)

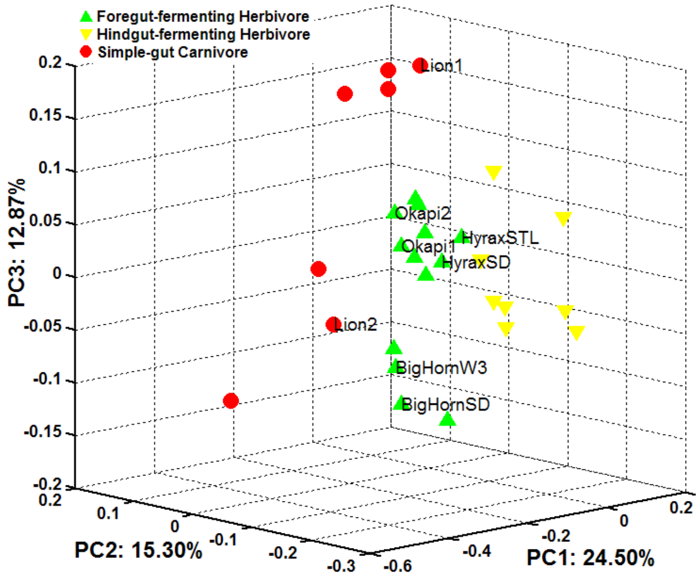

(b)

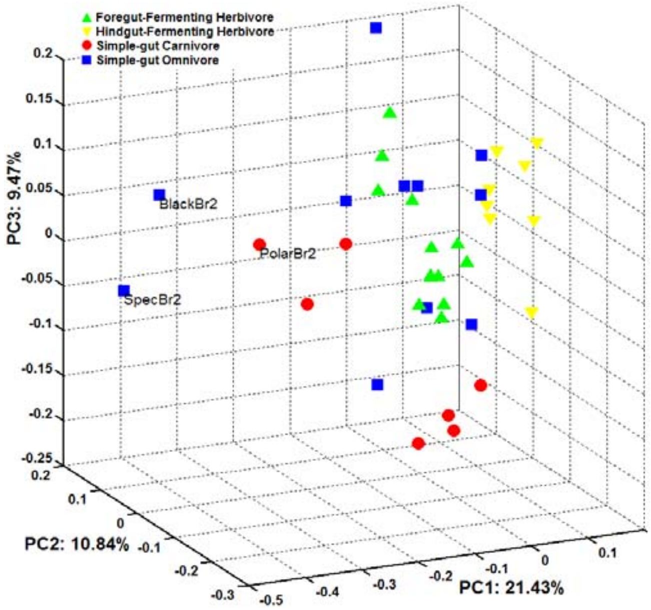

Figure S9 – Clustering of coastal water samples from different geographical locations.

(a) Geographical locations of coastal water samples from North American East Coast (n=9), Caribbean Sea (n=2), Eastern Tropical Pacific (n=1), Galapagos Islands (n=6) and Polynesia Archipelagos (n=1). (b) Clustering of samples based on 5-tuples and $d_2^S\ |M_0$ dissimilarity.

(a)

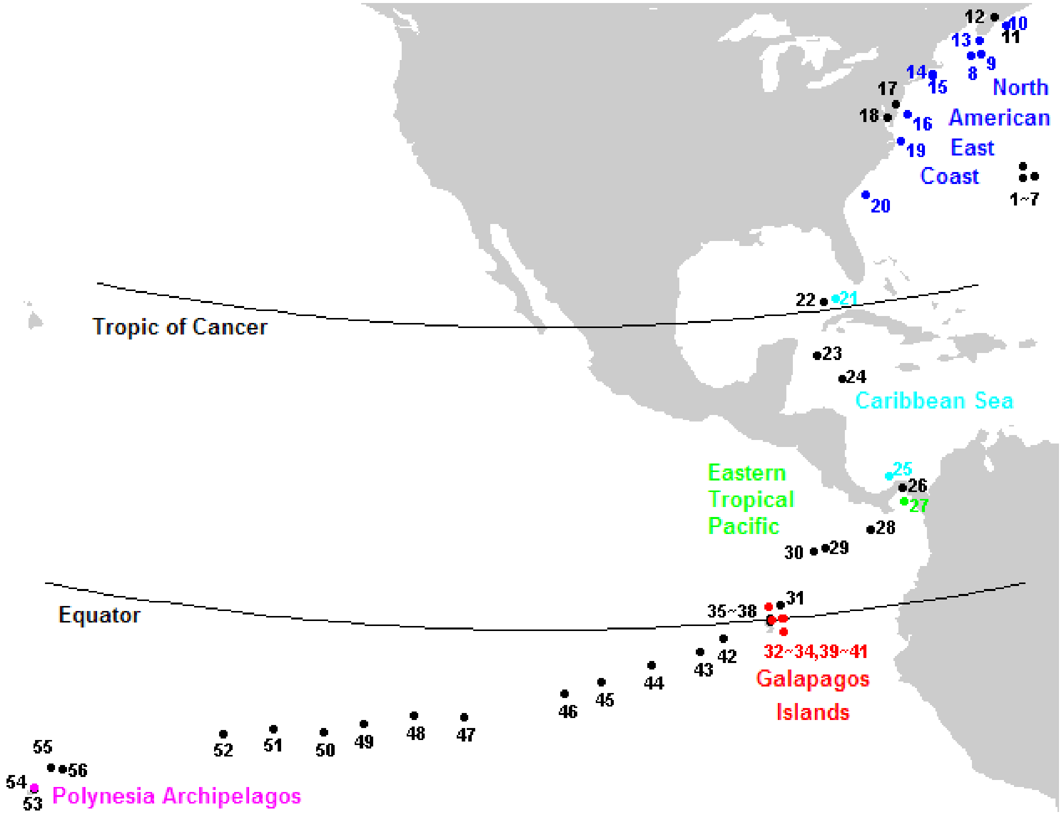

(b)

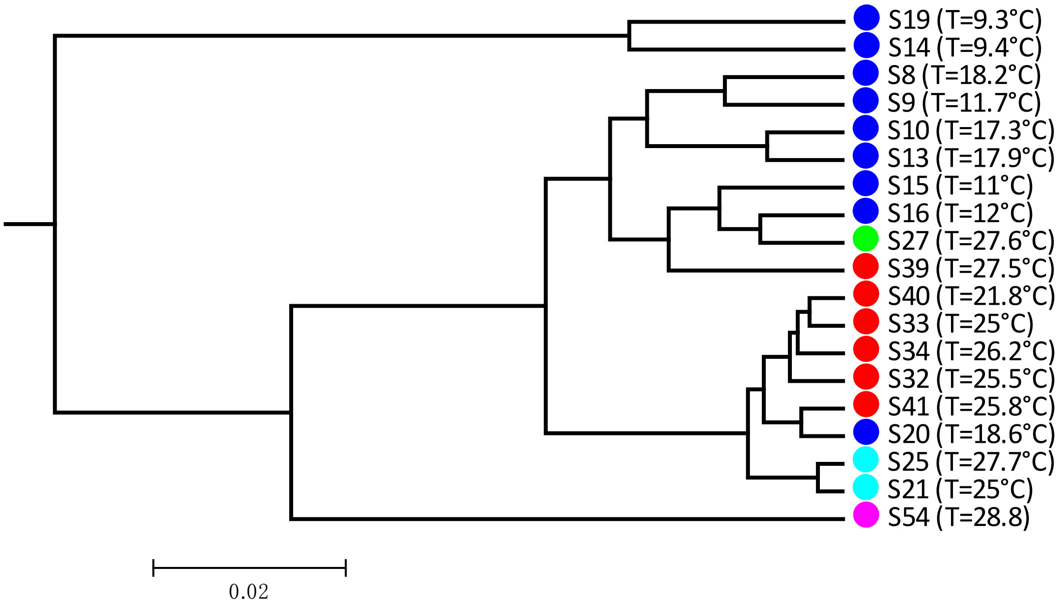

Figure S10 – Clustering and ordination analysis of 13 human gut metagenomic samples.

(a) Clustering tree based on 4-tuples and $d_2^S|M_0$. (b) Clustering tree based on 5-tuple and $d_2^S|M_0$. (c) PCoA plots based on 5-tuple and $d_2^S|M_0$: samples are labeled with age information in the left panel and are labeled with family information in the right panel.

(a)

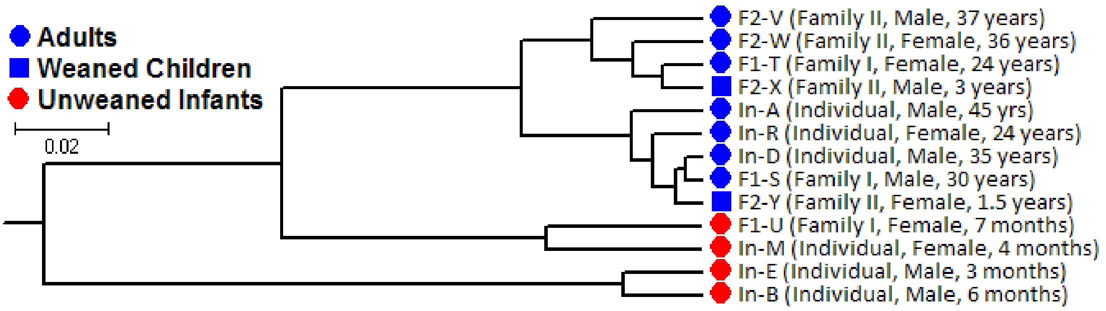

(b)

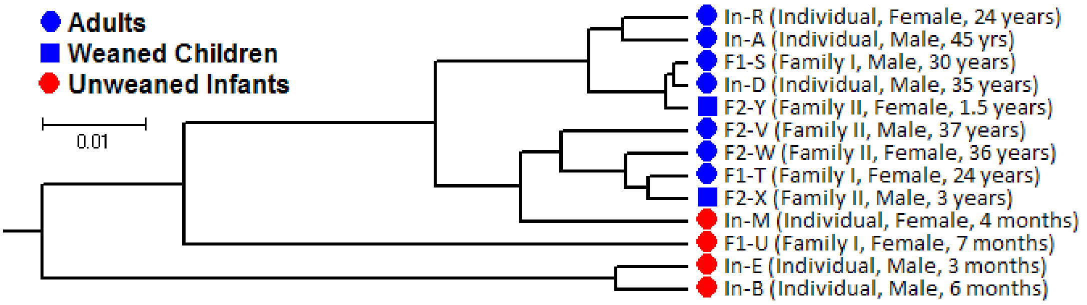

(c)

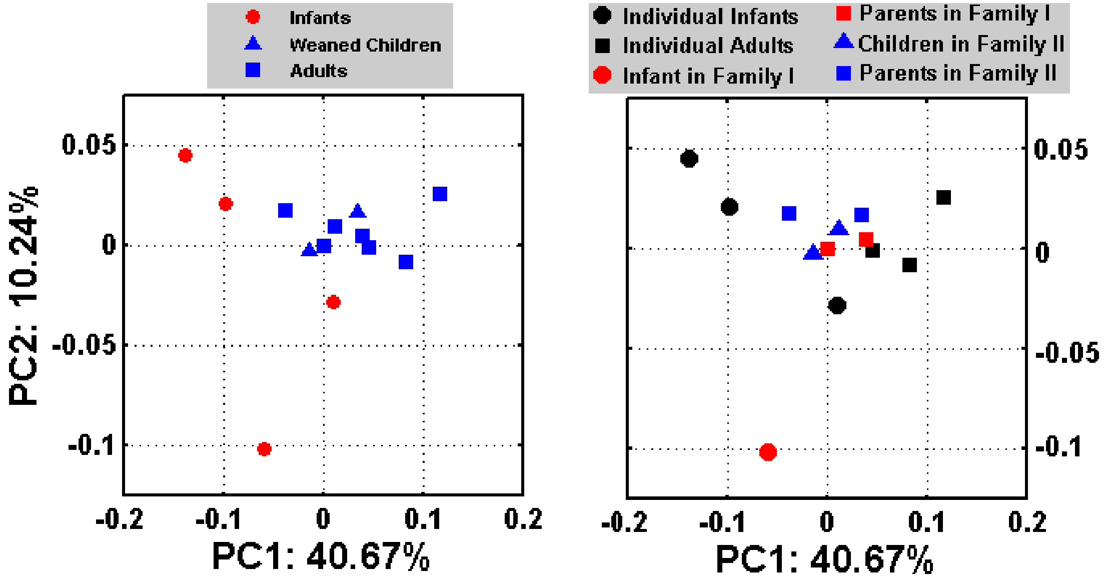

Figure S11 – Generation of 90 samples with group differences in species abundance of 5 bacteria species

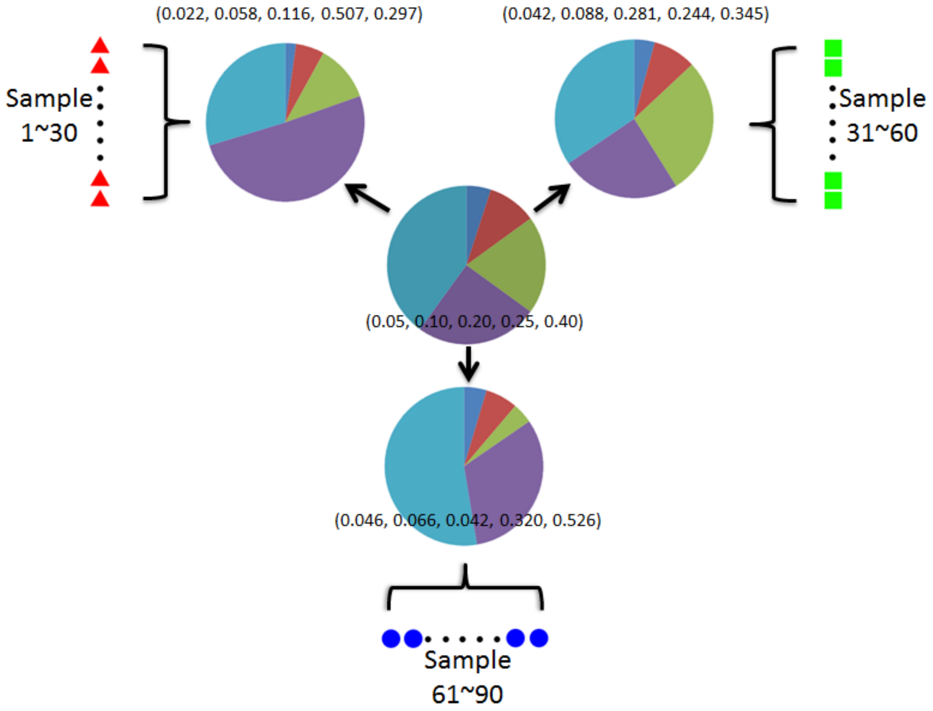

Figure S12 - Species abundance along the gradient location.

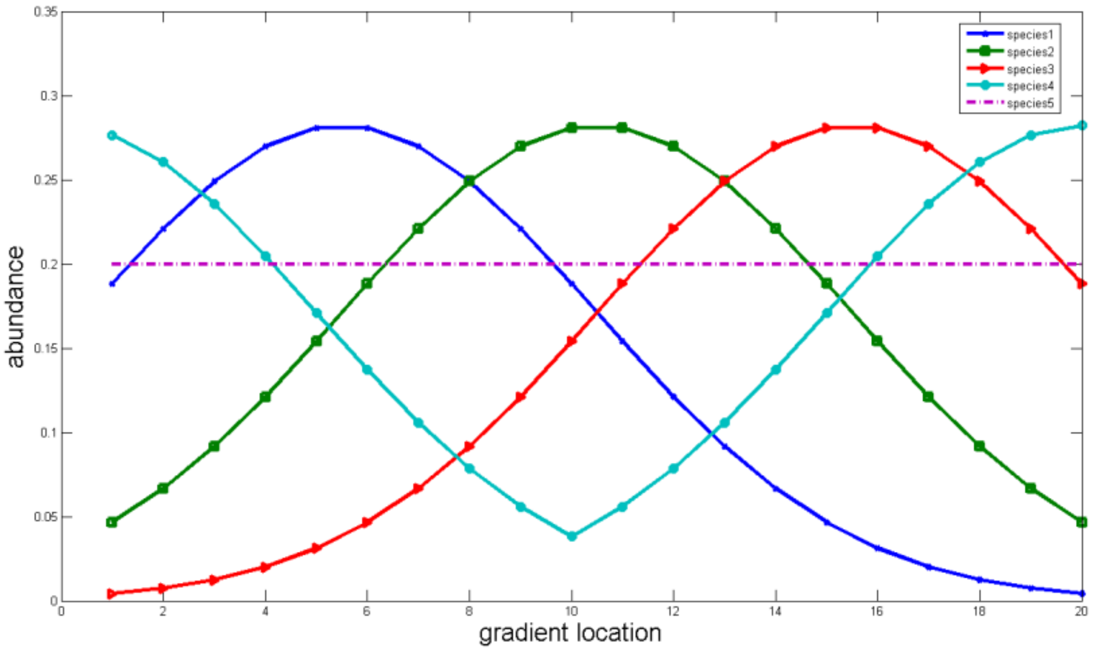

Table S1 – Summary of samples in the mammalian gut data set.

In the provenance column, SD, STL and W are short for San Diego Zoological Park, St. Louis Zoo and Wild, respectively.

SampleID	Common Name	Diet	Digestive Physiology	Provenance
AfElphSD3	African Elephant	Herbivore	Hindgut	SD
Armadillo	Armadillo	Carnivore	Simple-gut	STL
BaboonSTL	Baboon 1	Omnivore	Simple-gut	STL
BaboonW	Baboon 2	Omnivore	Simple-gut	W
BigHornSD	Bighorn Sheep 1	Herbivore	Foregut	SD
BigHornW3	Bighorn Sheep 2	Herbivore	Foregut	W
BlackBr2	Black Bear	Omnivore	Simple-gut	STL
BlackLemur	Black Lemur	Omnivore	Simple-gut	STL
BlackRhino1	Balck Rhinoceros	Herbivore	Hindgut	SD
BushDog1	Bush Dog	Carnivore	Simple-gut	STL
Callimicos	Callimicos	Omnivore	Simple-gut	STL
Capybara	Capybara	Herbivore	Hindgut	STL
Chimp1	Chimpanzee 1	Omnivore	Simple-gut	STL
Chimp2	Chimpanzee 2	Omnivore	Simple-gut	STL
Colobus	Colobus	Herbivore	Foregut	STL
Echidna	Echidna	Carnivore	Simple-gut	STL
Gazelle3	Gazelle	Herbivore	Foregut	STL
Giraffe2	Giraffe	Herbivore	Foregut	STL
GorillaSTL	Gorilla	Herbivore	Hindgut	STL
Horse1	Horse	Herbivore	Hindgut	W
Hyena2	Hyena	Carnivore	Simple-gut	STL
HyraxSD	Rock Hyrax 1	Herbivore	Foregut	SD
HyraxSTL	Rock Hyrax 2	Herbivore	Foregut	STL
Kroo3	Kangaroo	Herbivore	Foregut	STL
Lion1	Lion 1	Carnivore	Simple-gut	STL
Lion2	Lion 2	Carnivore	Simple-gut	STL
Okapi1	Okapi 1	Herbivore	Foregut	STL
Okapi2	Okapi 2	Herbivore	Foregut	STL
Orang1	Orangutan	Herbivore	Hindgut	STL
PolarBr2	Polar Bear	Carnivore	Simple-gut	STL
Rabbit	European Rabbit	Herbivore	Hindgut	STL
RTLemur	Ring-tailed Lemur	Omnivore	Simple-gut	STL
Saki	Saki	Omnivore	Simple-gut	STL
SecBr2	Spectacled Bear	Omnivore	Simple-gut	STL
SpgbkW	Springbok	Herbivore	Foregut	W
Squirrel	Squirrel	Omnivore	Simple-gut	STL
Urial2	Transcaspian Urial sheep	Herbivore	Foregut	STL
VWPig	Visayam Warty Pig	Herbivore	Foregut	SD
ZebraSTL1	Zebra	Herbivore	Hindgut	STL

Table S2 – Summary of samples in the global ocean data set.

Sample ID	Geographic Location	Sample Location	Habitat Type	Location	Water Depth (m)	T (°C)
JCVI_SMPL_1103283000001	Sargasso Sea	Sargasso Sea, Station 13	Open Ocean	31°2'06"n; 63°5'42"w	>4200	20
JCVI_SMPL_1103283000001	Sargasso Sea	Sargasso Sea, Station 11	Open Ocean	31°0'30"n; 64°9'27.6"w	>4200	20.5
JCVI_SMPL_1103283000002	Sargasso Sea	Sargasso Sea, Station 13	Open Ocean	31°2'06"n; 63°5'42"w	>4200	20
JCVI_SMPL_1103283000002	Sargasso Sea	Sargasso Sea, Station 11	Open Ocean	31°0'30"n; 64°9'27.6"w	>4200	20.5
JCVI_SMPL_1103283000003	Sargasso Sea	Sargasso Sea, Station 3	Open Ocean	32°0'29.4"n; 64°0'36.6"w	>4200	19.8
JCVI_SMPL_1103283000004	Sargasso Sea	Sargasso Sea, Station 13	Open Ocean	31°2'06"n; 63°5'42"w	>4200	20
JCVI_SMPL_1103283000005	Sargasso Sea	Sargasso Sea, Hydrostation S	Open Ocean	32°0'00"n; 64°0'00"w	>4200	22.9
JCVI_SMPL_1103283000006	Sargasso Sea	Sargasso Sea, Hydrostation S	Open Ocean	32°0'00"n; 64°0'00"w	>4200	22.9
JCVI_SMPL_1103283000007	Sargasso Sea	Sargasso Sea, Hydrostation S	Open Ocean	32°0'00"n; 64°0'00"w	>4200	22.9
JCVI_SMPL_1103283000008	North American East Coast	Gulf of Maine	Coastal	42°0'11"n; 67°4'24"w	106	18.2
JCVI_SMPL_1103283000009	North American East Coast	Browns Bank, Gulf of Maine	Coastal	42°1'10"n; 66°3'2"w	119	11.7
JCVI_SMPL_1103283000010	North American East Coast	Outside Halifax, Nova Scotia	Coastal	44°'14"n; 63°8'40"w	142	17.3
JCVI_SMPL_1103283000011	North American East Coast	Bedford Basin, Nova Scotia	Embayment	44°1'25"n; 63°8'14"w	64	15
JCVI_SMPL_1103283000012	North American East Coast	Bay of Fundy, Nova Scotia	Estuary	45°'42"n; 64°6'48"w	11	11.2
JCVI_SMPL_1103283000013	North American East Coast	Northern Gulf of Maine	Coastal	43°7'56"n; 66°0'50"w	139	17.9
JCVI_SMPL_1103283000014	North American East Coast	Newport Harbor, RI	Coastal	41°9'9"n; 71°1'4"w	12	9.4
JCVI_SMPL_1103283000015	North American East Coast	Block Island, NY	Coastal	41°'28"n; 71°6'8"w	32	11
JCVI_SMPL_1103283000016	North American East Coast	Cape May, NJ	Coastal	38°6'24"n; 74°1'6"w	10	12
JCVI_SMPL_1103283000017	North American East Coast	Delaware Bay, NJ	Estuary	39°5'4"n; 75°0'15"w	8	11
JCVI_SMPL_1103283000018	North American East Coast	Chesapeake Bay, MD	Estuary	38°6'49"n; 76°5'2"w	25	1
JCVI_SMPL_1103283000019	North American East Coast	Off Nags Head, NC	Coastal	36°'14"n; 75°3'41"w	20	9.3
JCVI_SMPL_1103283000020	North American East Coast	South of Charleston, SC	Coastal	32°0'25"n; 79°5'50"w	31	18.6

JCVI_SMPL_1103283000021	Caribbean Sea	Off Key West, FL	Coastal	24°9'18"n; 83°'12"w	47	25
JCVI_SMPL_1103283000022	Caribbean Sea	Gulf of Mexico	Coastal Sea	24°0'29"n; 84°0'40"w	3333	26.4
JCVI_SMPL_1103283000023	Caribbean Sea	Yucatan Channel	Open Ocean	20°1'21"n; 85°4'49"w	4513	27
JCVI_SMPL_1103283000024	Caribbean Sea	Rosario Bank	Open Ocean	18°'12"n; 83°7'5"w	4470	27.4
JCVI_SMPL_1103283000025	Caribbean Sea	Northeast of Col	Coastal	10°2'59"n; 80°5'16"w	3336	27.7
JCVI_SMPL_1103283000026	Panama Canal	Lake Gatun	Fresh Water	9°'52"n; 79°0'10"w	4.2	28.6
JCVI_SMPL_1103283000027	Eastern Tropical Pacific	Gulf of Panama	Coastal	8°'45"n; 79°1'28"w	76	27.6
JCVI_SMPL_1103283000028	Eastern Tropical Pacific	250 miles from Panama City	Open Ocean	6°9'34"n; 82°4'14"w	2431	29.3
JCVI_SMPL_1103283000029	Eastern Tropical Pacific	30 miles from Cocos Island	Open Ocean	5°8'24"n; 86°3'55"w	1139	28.7
JCVI_SMPL_1103283000030	Eastern Tropical Pacific	Dirty Rock, Cocos Island	Fringing Reef	5°3'10"n; 87°'16"w	30	28.3
JCVI_SMPL_1103283000031	Galapagos Islands	134 miles NE of Galapagos	Open Ocean	1°5'51"n; 90°7'42"w	2386	27.8
JCVI_SMPL_1103283000032	Galapagos Islands	Devil's Crown, Floreana Island	Coastal	1°2'58"s; 90°5'22"w	2.3	25.5
JCVI_SMPL_1103283000033	Galapagos Islands	Coastal Floreana	Coastal	1°3'1"s; 90°9'11"w	156	25
JCVI_SMPL_1103283000034	Galapagos Islands	North James Bay, Santigo Island	Coastal	0°2'0"s; 90°0'7"w	12	26.2
JCVI_SMPL_1103283000035	Galapagos Islands	Warm seep, Roca Redonda	Warm Seep	0°6'20"n; 91°8'0"w	19	26.9
JCVI_SMPL_1103283000036	Galapagos Islands	Upwelling, Fernandina Island	Coastal upwelling	0°8'4"s; 91°9'6"w	19.6	18.6
JCVI_SMPL_1103283000037	Galapagos Islands	Mangrove on Isabella Island	Mangrove	0°5'38"s; 91°'10"w	1.6	25.4
JCVI_SMPL_1103283000038	Galapagos Islands	Punta Cormorant, Hypersaline Lagoon, Floreana Island	Hypersaline	1°3'42"s; 90°5'45"w	0.3	37.6
JCVI_SMPL_1103283000039	Galapagos Islands	North Seamore Island	Coastal	0°2'59"s; 90°6'47"w	35	27.5
JCVI_SMPL_1103283000040	Galapagos Islands	Wolf Island	Coastal	1°3'21"n; 91°9'1"w	71	21.8
JCVI_SMPL_1103283000041	Galapagos Islands	Cabo Marshall, Isabella Island	Coastal	0°'15"s; 91°1'52"w	67	25.8
JCVI_SMPL_1103283000042	Eastern Tropical Pacific	Equatorial Pacific TAO Buoy	Open Ocean	1°8'26"s; 95°'53"w	3334	28
JCVI_SMPL_1103283000043	Tropical South Pacific	Tropical South Pacific	Open Ocean	2°4'55"s; 97°1'5"w	>4000	28.4
JCVI_SMPL_1103283000044	Tropical South Pacific	Tropical South Pacific	Open Ocean	3°0'36"s; 101°2'26"w	>4000	28.6

JCVI_SMPL_1103283000045	Tropical South Pacific	Tropical South Pacific	Open Ocean	4°9'56"s; 105°4'12"w	>4000	27.8
JCVI_SMPL_1103283000046	Tropical South Pacific	Tropical South Pacific	Open Ocean	5°5'48"s; 108°1'13"w	>4000	28
JCVI_SMPL_1103283000047	Tropical South Pacific	Tropical South Pacific	Open Ocean	7°6'27"s; 116°7'9"w	>4000	27.6
JCVI_SMPL_1103283000048	Tropical South Pacific	Tropical South Pacific	Open Ocean	7°9'40"s; 120°4'8"w	>4000	27.6
JCVI_SMPL_1103283000049	Tropical South Pacific	600 miles from F. Polynesia	Open Ocean	8°4'54"s; 124°4'23"w	>4000	27.6
JCVI_SMPL_1103283000050	Tropical South Pacific	400 miles from F. Polynesia	Open Ocean	9°1'3"s; 127°6'2"w	>4000	28.3
JCVI_SMPL_1103283000051	Tropical South Pacific	300 miles from F. Polynesia	Open Ocean	9°4'16"s; 131°9'30"w	>4000	28.7
JCVI_SMPL_1103283000052	Tropical South Pacific	201 miles from F. Polynesia	Open Ocean	10°'53"s; 135°6'58"w	2400	28.6
JCVI_SMPL_1103283000053	Polynesia Archipelagos	Moorea, Cooks Bay	Coral Reef	17°8'33"s; 149°8'44"w	34	28.9
JCVI_SMPL_1103283000054	Polynesia Archipelagos	Moorea, Outside Cooks Bay	Coastal	17°7'11"s; 149°7'56"w	900	28.8
JCVI_SMPL_1103283000055	Polynesia Archipelagos	Tikehau Lagoon	Coral Atoll	15°6'40"s; 148°3'28"w	24	27.8
JCVI_SMPL_1103283000056	Polynesia Archipelagos	Rangirora Atoll	Coral Reef Atoll	15°'37"s; 147°6'6"w	10	27.3

Table S3 - Parsimony scores on the clustering tree of 19 coastal water samples given by k-tuple method.

Monte Carlo p-value was estimated by comparing observed parsimony score to the scores in 10000 randomly joined trees: parsimony score=5, p=0.001; parsimony score=6, p=0.008; parsimony score=7, p=0.067; parsimony score=8, p=0.306.

k	2	3	4	5	6	7	8	9	10
d_2	8	8	7	6	6	6	6	6	7
$d_2^S \mid M_0$	6	6	6	6	6	6	6	6	6
$d_2^S \mid M_1$	6	6	6	6	6	7	6	6	6
$d_2^S \mid M_2$	NA	6	6	6	6	7	6	6	6
$d_2^S \mid M_3$	NA	NA	5	6	6	6	6	6	6
$d_2^* \mid M_0$	6	6	6	6	6	6	6	7	7
$d_2^* \mid M_1$	7	6	6	6	7	6	7	7	7
$d_2^* \mid M_2$	NA	6	6	6	7	7	7	7	7
$d_2^* \mid M_3$	NA	NA	6	6	6	7	7	7	7
Ma	8	8	8	6	6	6	6	6	6
Eu	8	8	8	7	7	6	6	7	7
Ch	7	8	8	8	7	7	7	7	7
Hao	NA	6	6	8	7	6	7	7	6
Willner	9	8	8						

Table S4 – Summary of samples in the human gut data set.

Sample Status	Sample name	Gender	Age
Individual	In-A	Male	45 years
Individual	In-B	Male	6 months
Individual	In-D	Male	35 years
Individual	In-E	Male	3 months
Individual	In-M	Female	4 months
Individual	In-R	Female	24 years
Family I	F1-S	Male	30 years
	F1-T	Female	28 years
	F1-U	Female	7 months
Family II	F2-V	Male	37 years
	F2-W	Female	36 years
	F2-X	Male	3 years
	F2-Y	Female	1.5 years

Table S5 - Parsimony scores on the clustering tree of 13 human gut sample between 4 unweaned infants and 9 adults or children given by sequence signature methods.

Monte Carlo p-values were estimated by comparing the observed parsimony score with the parsimony scores in 10,000 randomly joined trees: parsimony score=1, p=0.005; parsimony score=2, p=0.05; parsimony score=3, p=0.36.

k	2	3	4	5	6	7	8	9	10
d_2	3	3	2	2	2	2	2	2	2
$d_2^s \mid M_0$	3	1	1	2	2	2	2	1	1
$d_2^s \mid M_1$	3	1	1	1	1	1	1	1	1
$d_2^s \mid M_2$	NA	2	1	1	1	1	1	1	1
$d_2^s \mid M_3$	NA	NA	1	1	1	1	1	1	1
$d_2^* \mid M_0$	3	1	1	2	2	2	1	1	1
$d_2^* \mid M_1$	3	1	1	1	1	1	1	1	1
$d_2^* \mid M_2$	NA	1	1	1	1	1	1	1	1
$d_2^* \mid M_3$	NA	NA	1	1	1	1	1	1	1
Hao	NA	2	1	1	1	1	1	1	1
Ma	3	3	2	2	2	2	2	2	2
Eu	3	3	2	2	2	2	2	2	2
Ch	3	3	3	2	2	2	2	2	3
Willner	4	4	4						
